# Living in a pandemic: changes in mobility routines, social activity and adherence to COVID-19 protective measures

**DOI:** 10.1038/s41598-021-04139-1

**Published:** 2021-12-27

**Authors:** Lorenzo Lucchini, Simone Centellegher, Luca Pappalardo, Riccardo Gallotti, Filippo Privitera, Bruno Lepri, Marco De Nadai

**Affiliations:** 1 Fondazione Bruno Kessler (FBK), Trento, Italy; 2 Institute of Information Science and Technologies, National Research Council (ISTI-CNR), Pisa, Italy; 3 Cuebiq Inc., New York, NY USA

**Keywords:** Computational science, Public health

## Abstract

Non-Pharmaceutical Interventions (NPIs), aimed at reducing the diffusion of the COVID-19 pandemic, have dramatically influenced our everyday behaviour. In this work, we study how individuals adapted their daily movements and person-to-person contact patterns over time in response to the NPIs. We leverage longitudinal GPS mobility data of hundreds of thousands of anonymous individuals to empirically show and quantify the dramatic disruption in people’s mobility habits and social behaviour. We find that local interventions did not just impact the number of visits to different venues but also how people experience them. Individuals spend less time in venues, preferring simpler and more predictable routines, also reducing person-to-person contacts. Moreover, we find that the individual patterns of visits are influenced by the strength of the NPIs policies, the local severity of the pandemic and a risk adaptation factor, which increases the people’s mobility regardless of the stringency of interventions. Finally, despite the gradual recovery in visit patterns, we find that individuals continue to keep person-to-person contacts low. This apparent conflict hints that the evolution of policy adherence should be carefully addressed by policymakers, epidemiologists and mobility experts.

## Introduction

The COVID-19 pandemic has prompted many countries to implement a diverse set of Non-Pharmaceutical Interventions (NPIs) such as international travel restrictions, physical distancing mandates, closures of business venues, and stay-at-home orders to prevent the spread of the virus^1–7^. These policies have had a profound impact on numerous aspects of human life including employment^8,9^, economy^10–13^ and people’s social behaviour^14–17^.

In this context, mobile phone data offered unprecedented opportunities to capture the effects of the NPIs and to understand better their impact on the evolution of the epidemic^18,19^. Different technological and telecommunication companies have released analysis and data to help researchers estimate the epidemic spread^16,20–25^, mobility reduction^16,26–29^, physical distancing^30,31^, physical activity^32^, and informing on the efficacy of NPIs^25,33–36^. However, most of these works are based on aggregated data and focus their analysis on macro mobility indicators to inform epidemic models^16,31,34^.

While the short term behavioural adaptation to NPIs and their impact on epidemic models is of paramount importance to promptly respond to the pandemic threat, an in-depth understanding of how individual behaviour adapted over time is still lacking. Understanding the long term behaviour in a condition of sustained epidemic threat represents an essential factor in facilitating policy adherence.

This paper studies the changes in the daily routinary behaviour of people, focusing on how individuals adapted their pattern of visits and social contacts over time. We combine Point Of Interest (POI) information extracted from OpenStreetMap (OSM) with privacy-enhanced longitudinal GPS mobility traces of more than 837,000 anonymous opted-in individuals, measured for nine months from 3 January 2020 to 1 September 2020. Our dataset has an average accuracy of 22 m and covers 16 hours of activity per day. This high precision and coverage allow us to examine human mobility at a fine spatial and temporal granularity while ensuring users’ privacy (see SI Section S2-A for additional details). We analyse, model, and compare individual’s mobility in four US states, including those with the highest and lowest values of daily COVID-19 death rate and NPIs stringency: Arizona (many deaths and low stringency), Oklahoma (few deaths and low stringency), Kentucky (few deaths and high stringency), and New York (many deaths and high stringency) (see SI Section S5 for details).

Our results describe the disruption effect of the COVID-19 pandemic on human behaviour over time at an unprecedented level of detail. Overall, we find that people changed both how they visit places and how they experience them. They spend less time in venues, preferring simpler and more predictable routines. Individual patterns of visits to POIs are shaped by the strength of the NPIs policies, the pandemic’s local severity and a risk adaptation factor, which increases the people’s mobility regardless of the stringency of interventions. Finally, despite the gradual recovery in visit patterns, person-to-person contacts remained lower than expected given the increased time spent at venues.

## Results

**Figure 1:**
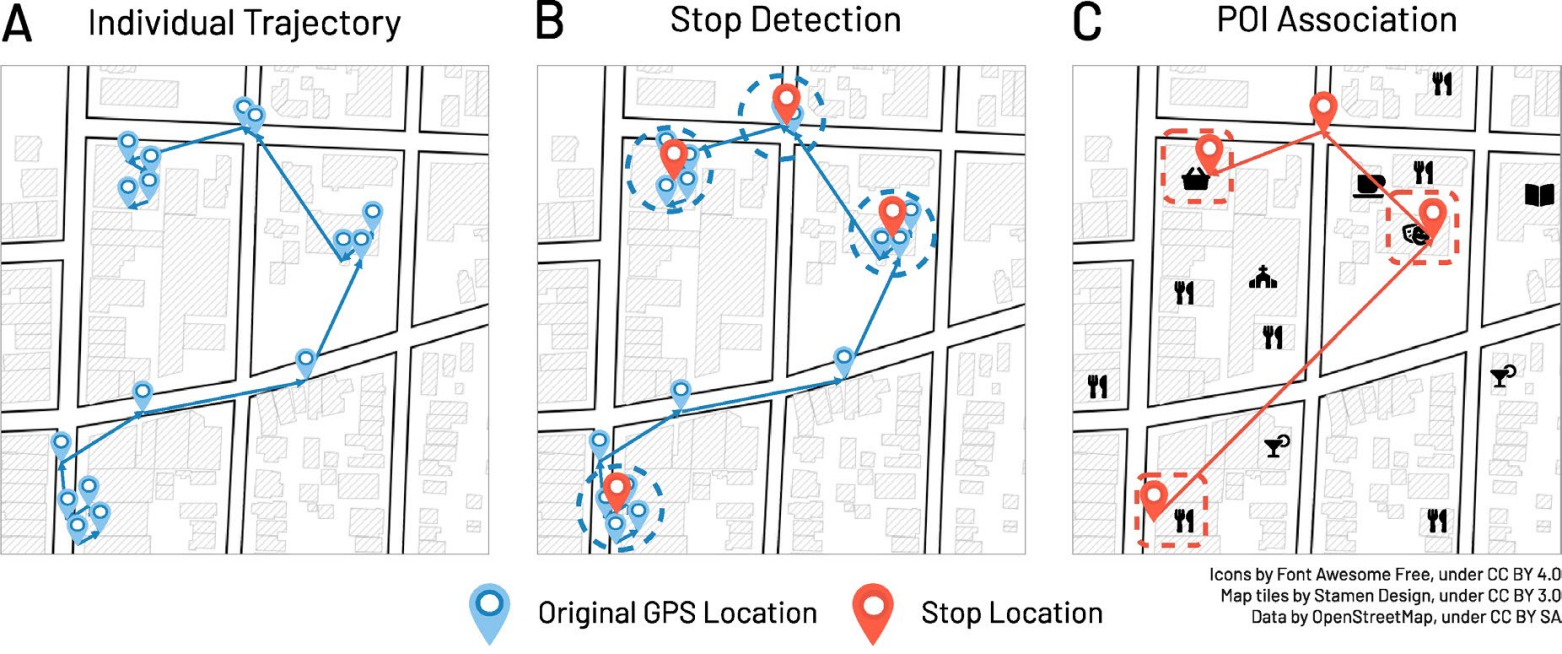
(**A**) From an individual’s original GPS trajectory, we detect a stop whenever the individual spends at least 5 minutes within a distance of 65 meters from a given trajectory point. (**B**) We detect stop locations through a combination of the Lachesis^37^ and DBSCAN algorithms^38^. (**C**) If present, we associate each stop location to the nearest Point Of Interest (POI) within a distance of 65 m.

We explore and characterize human mobility from an individual’s set of stop locations, defined as places where a person stays for at least 5 minutes within a distance of 65 meters. From the original GPS data (see Fig. 1A), we detect the stop locations of each individual through a combination of the Lachesis^37^ and DBSCAN algorithms^38^ (see “Methods” and Fig. 1B). As a result, stop locations are described as tuples (*lat, lon, start-time, end-time*), where an individual stays in a particular location with latitude (*lat*) and longitude (*lon*) from *start-time* to *end-time*. *lat* and *lon* are the mean latitude and longitude values of the GPS points found within the specified distance of 65 meters (we refer to the “Methods” and SI Section S3 for the details). Then, we focus on the home and work locations of people. To preserve privacy, the data provider obfuscates users’ precise home and work locations by transforming it to the centroid of the corresponding Census Block Group. Thus, we identify the home census block group of users, from now on called *Residential* area, by looking at the most visited locations during the nights (from 8 pm to 4 am) with a moving time window of 28 days. Similarly, we also identify the *Workplace*, defined as the most visited census block group during the week (from 9 am to 5 pm), which is not marked *Residential*. We refer to SI Section S4 for additional details.

Finally, we add semantic meaning to individuals’ mobility trajectories associating each stop location to the nearest Point of Interest (POI), extracted from OpenStreetMap (OSM)^39^, whenever a POI lies within 65 m from the stop location (see Fig. 1C). POIs are commonly described as public locations that people may find interesting, for example, for business or recreational activities^40^. Since the OSM POIs taxonomy is not hierarchically organized, we create a human-curated mapping from the OSM tagging system^41^ to the Foursquare venue category hierarchy^42^ (see “Methods” and SI Section S7 for details). SI Section S7-B shows the popularity of POIs in different states, highlighting the variety of the visiting behaviour in the US.

To validate the data provided by Cuebiq and our pre-processing, we compute the correlation of the time-series of visits to POIs, residential areas and workplace areas between our data, Google data^26^, and Foursquare data^43^. We report an average Pearson correlation in New York state of 0.91 and an average Pearson correlation of 0.84 in all four selected states (see SI Section S8).

Starting from the visits of each individual we analyse how people’s lifestyle adapted during the pandemic.

### Changes in POIs visit patterns

We begin our analysis by describing the impact of the COVID-19 pandemic on the visits to POIs over time. Overall, we observe that NPIs are associated with fewer visits to POIs, confirming previous results obtained with similar datasets^26,32,34^.

**Figure 2:**
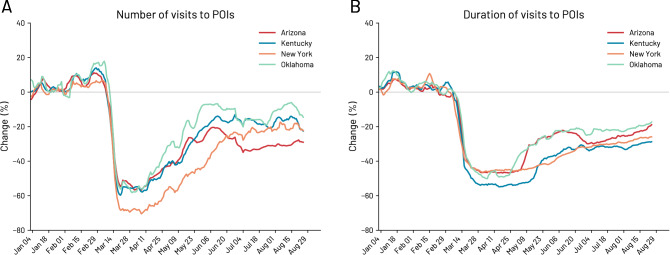
Changes in number and duration of visits to all POIs in the states of Arizona, Kentucky, New York and Oklahoma. (**A**) Percentage change over time in the number of visits with respect to the baseline period (3 January 2020–28 February 2020) for all types of venues. (**B**) Change in percentage of the duration of visits over time with respect to the baseline period of the median duration of visits to all POIs. For visualization purposes, the original curves are smoothed using a rolling average of seven days.

Figure 2 shows how the number of visits (panel A) to POIs and the duration of those visits change over time in the four selected US states. It also highlights the differences across states in which the epidemiological situation and the stringency of the enacted policies differed most (see Section S5 for more details). For example, the state of New York experienced the most significant drop in the number of visits to all types of POIs (for details on POIs refer to SI Section S7) with a reduction of $69\%$. The other states experienced smaller but similar reductions in POIs visits in a range between $55\%$ and $60\%$ (see SI Section S7-E for a more detailed analysis on single POI categories). Noteworthy differences are present also in the recovery phases due to the different re-opening measures. As an example, due to early re-opening phases and the absence of restrictions after the end of the stay-at-home order on 15 May 2020, the state of Arizona experienced a smaller drop in the number of visits to POIs with respect to New York state (Fig. 2A). However, in the long run, the state of New York recovers better since Arizona was forced to introduce new closures due to rising COVID-19 cases. Interestingly, we observe that individuals allocate the everyday time spent similarly across states (Fig. 2B), regardless of the NPIs implemented, but again we find some differences potentially due to the different NPI strategies. We refer to SI Section S9 for a set of additional metrics (i.e. individuals’ unique stop locations, diversity of visits, and radius of gyration) supporting our findings.

**Figure 3:**
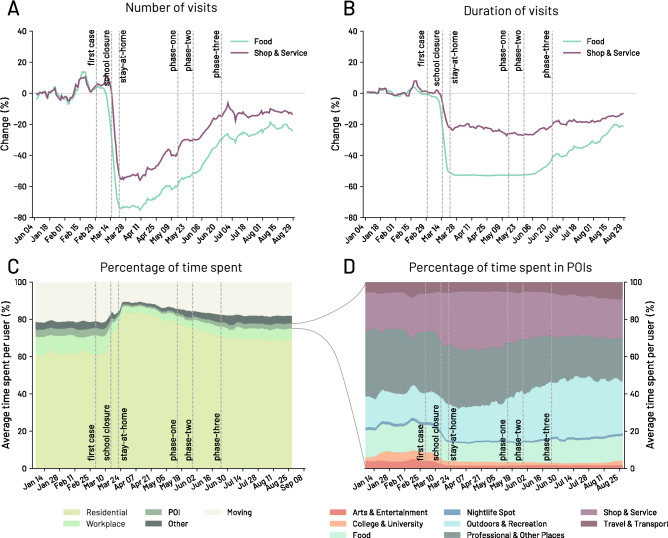
Changes in number and duration of visits to POIs in the state of New York. (**A**) Percentage change over time in the number of visits with respect to the baseline period (3 January 2020–28 February 2020) for venues in the *Food* and *Shop & Service* categories. (**B**) Change in percentage of the duration of visits over time with respect to the baseline period of the median duration of visits to *Food* and *Shop & Service* POIs. For visualization purposes, the original curves are smoothed using a rolling average of seven days. Vertical dashed lines indicate the date of restrictions and orders imposed by the state of New York government. (**C**) Percentage of time spent by people at *Residential*, *Work*, *POIs*, *Other*, and *Moving* (i.e. people in movement). (**D**) Percentage of time spent by people in venues under the eight first-level categories of POIs.

A similar change in mobility behaviour is also found at a more fine-grained level, by focusing on the different POI categories. As shown in Fig. 3, in the state of New York, POIs belonging to the *Food* (e.g., restaurants) and *Shop & Service* (e.g., book stores and supermarkets) categories experienced low points of $-75\%$ and $-56\%$ in the number of visits, respectively. The duration of visits is also severely impacted by COVID-19 reaching low points of $-53\%$ and $-27.3\%$ for *Food* and *Shop & Service*, respectively. These reductions in the number of visits are heterogeneous. For example, essential shops such as supermarkets faced a lower reduction in the number of visits than the non-essential shops (with low points of $-38.3\%$ vs $-67\%$ shortly after the stay-at-home order) and even increased the number of visits before the stay-at-home order (see SI Section S7-F).

Since two weeks after the stay-at-home order, we observe an increase in the number of visits, which comes after the government’s decision of expanding the list of the essential businesses and the progressive relaxation of government restrictions (see Fig. 3A,B) throughout the various re-opening phases, conveniently named phase-one, phase-two, and phase-three (see the “Methods” Section). Interestingly, while the number of visits gradually recover in the period between 25 March 2020 and 20 May 2020 (see Fig. 3A), the duration of visits recover much slowly. At the end of the period of study, the duration of visits increases by 60% and 51% for *Food* and *Shop & Service* respectively from the pre-pandemic period, while visits recover by 69% and 67% for *Food* and *Shop & Service* respectively from the pre-pandemic period. Thus, individuals changes how they experience POIs. We note that the number of visits to *Food* and *Shop & Service* starts increasing between the stay-at-home order and phase-one (see Fig. 3A), despite the absence of significant changes in the restrictions. We will later analyse this finding.

Figure 3C shows the distribution of time spent by individuals over time in the state of New York. We group the time spent into five categories: *Residential*, *Workplace*, *POI*, *Other* (i.e. stop locations not matched with a POI and not detected neither as *Residential* or *Workplace*), and *Moving* (i.e. time spent moving from place to place). After the stay-at-home order, we find that the percentage of time spent in residential areas increases significantly to the expense of the time spent at *Workplace*, *POI*, *Other*, and *Moving*. After phase three, we find that the time spent in residential areas gradually reduces while other categories increase. The time spent in each category, however, does not return to the pre-pandemic period.

Figure 3D shows the percentage of time spent by people in the venues of the eight first-level categories of POIs. The time spent at *Shop & Service* increases at the expense of the other POIs. Then, after phase three, people significantly increase the time spent in some categories such as *Outdoors & Recreation* and *Travel & Transport*, probably also due to seasonal effects, but other categories such as *Arts & Entertainment* do not recover to pre-pandemic levels. We refer the readers to SI Section S1 for a more complete example of the reduction of visits in New York City.

### Disruption of individual mobility routines

By looking at the aggregated mobility, we only have a partial view of the disruption in people’s lives due to COVID-19. Individual’s behaviour exhibit mobility motifs and routines that characterize the chronological sequence of where they go, spend money and meet people^44^. For this reason, we now focus on the chronological sequences of visits, which help us understand the complexity of changes in mobility.

We transform the individual’s chronological sequence of visits to places into a sequence of symbols (e.g., *Food*, *Residential*, *Workplace*). Then, we apply the Sequitur algorithm^44–46^ to generate a hierarchical representation of the original sequence, compressing repeated occurrences. Due to computational constraints, we focus on two 4-week periods, before (from 1 February 2020 to 28 February 2020) and during pandemic (from 21 March 2020 to 17 April 2020) and focus on significant routines of each user. To detect the significant routines, we generate 1000 randomized sequences with the same number of visits as the original sequence. Then, we define an individual’s sequence as significant if it has a z-score $<2$ between the occurrence of the real routines with respect to the randomized ones. Finally, we filter out all the non-significant routines. We refer to the “Methods” for additional details.

**Figure 4:**
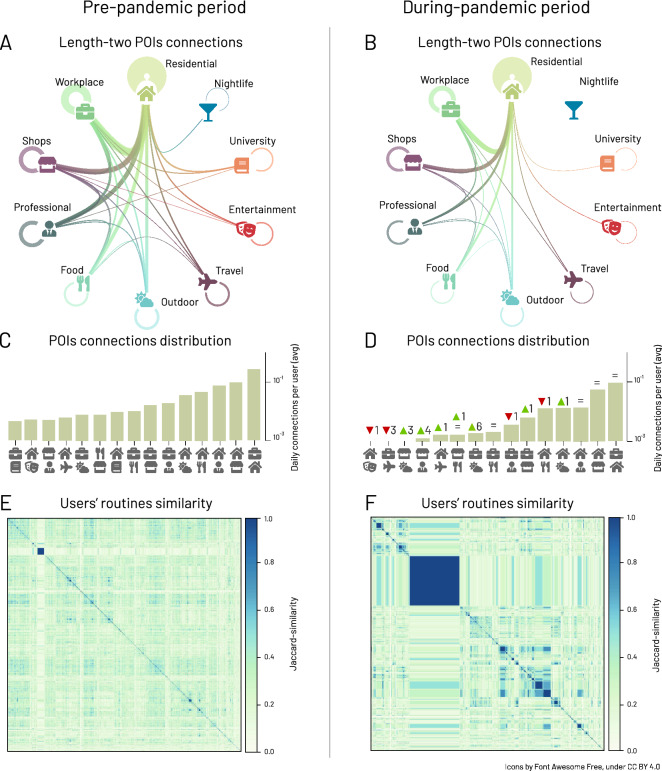
Changes in routine behaviour before and during the pandemic periods. (**A**, **B**) Network of the subsequent movements between POI categories for all users in the state of New York before and during the pandemic. The thickness of the links is proportional to the square root of the number of movements between the two POI categories. For visualization purposes we are showing only links for which the average number of daily movements is greater than $1/10\,000$. Pre-pandemic (**C**) and during-pandemic (**D**) distributions of these intensities excluding self-loops (e.g., *Residential* $\leftrightarrow$ *Residential*, *Food* $\leftrightarrow$ *Food* connections). Jaccard similarity matrix between individuals’ routines before the pandemic (**E**) and during the pandemic (**F**).

The significant routines represent meaningful sub-sequences of an individual’s mobility and allow to better understand, at a micro-level, how human mobility preferences changed with COVID-19. To that end, we model the ordered sequences of visits to POIs as a weighted undirected network in which the nodes represent the POI categories, and a link between category $c_1$ and category $c_2$ exists if there is at least a chronological sequence where $c_1$ immediately follows $c_2$ or vice-versa. The weight of the link represents the daily average proportion of sequences containing $[c_1, c_2]$ or $[c_2, c_1]$. Figure 4A,B shows the weighted network of the POI categories in the state of New York, where the size of the links is proportional to the intensity of the relationship between the two POI categories. We observe that all links reduce their intensity (on average − 79%) with the exception of the *Residential* $\leftrightarrow$ *Residential*, which increased by 5%.

Figure 4C and D shows the distributions of the network weights in the pre-pandemic (from 1 February 2020 to 28 February 2020) and during pandemic (from 21 March 2020 to 17 April 2020) periods in New York state, from which we excluded all self-loops (e.g., *Residential* $\leftrightarrow$ *Residential*, *Food* $\leftrightarrow$ *Food* connections). We observe the mildest reduction for *Shop & Service* $\leftrightarrow$ *Residential* (− 40% visits), *Shop & Service* $\leftrightarrow$ *Workplace* (− 61%), and *Outdoors & Recreation* $\leftrightarrow$ *Residential* (− 63%), which might be interpreted as essential routines. Higher levels of reduction are found in those POIs connections which include sectors strongly afflicted by NPIs: *Arts & Entertainment* $\leftrightarrow$ *College & University* (− 95%), *College & University* $\leftrightarrow$ *Nightlife Spot* (− 95%), *College & University* $\leftrightarrow$ *Residential* (− 93%), and *Arts & Entertainment* $\leftrightarrow$ *Food* (− 92%) (for further details refer to SI Fig. S20).

The dramatic change of people’s behaviour also emerges from the similarity between the characteristic routine of different individuals. We represent each individual’s significant routine behaviour regarding the presence or absence of two-elements sequences between POI categories. Then, we compute the Jaccard similarity between a randomly selected sample of 10,000 individuals. Finally, we apply agglomerative hierarchical clustering^47^ to find relevant groups of individuals with similar routine behaviour. By comparing Fig. 4E and F, we observe that Fig. 4F contains larger clusters, which means that mobility routines simplify and people’s behaviour gets more homogeneous. This result is also quantitatively confirmed by the larger silhouette score^48^ of all individuals during-pandemic period compared with the pre-pandemic period (see SI Section S10-D for details). By inspecting the everyday routines in the two biggest clusters, which include almost 34% of users, we observe that individuals limit their mobility to *Residential* $\leftrightarrow$ *Residential*, *Residential* $\leftrightarrow$*Shops & Services* and *Shops & Services* $\leftrightarrow$ *Shops & Services* routines. We refer to SI Section S10-E for a comparison between the six biggest clusters.

During the pandemic period, we also find that individuals tend to favour simpler and more redundant sequences. We measure the compression ratio^44^ defined as the length of the original sequence divided by the length of the Sequitur compressed sequence. On average, in the state of New York, the individual’s sequences before the pandemic have a compression ratio of 2.75, while during the COVID-19 pandemic, the compression ratio increases by 40%, reaching 3.79 (see SI Fig. S16). Similar results apply in all the other states (see SI Fig. S10B).

### Evolution of policy adherence and risk adaptation in visit patterns

In Fig. 3A, we observe that the number of visits starts increasing between the stay-at-home order and phase-one, despite the absence of significant changes in the restrictions. To explain this behaviour, we hypothesise the presence of progressive behavioural relaxation and adaptation to the epidemic risk, also observed by previous literature^30,49^ (and sometimes called “pandemic fatigue"^50^).

We use a multivariate Bayesian linear mixed model to examine the daily number of visits to POIs in each state through the strength of the NPIs, daily death ratio, and weather conditions (i.e. daily max temperature and precipitations). NPIs’ strength and daily death ratio are used as proxies to capture the local socio-epidemiological condition. More precisely, the first include information about the intensity of restrictions, such as closures and gathering restrictions, while the second quantify the severity of the condition and the local burden of the disease over time. Similarly, weather conditions account for environmental confounding variables which might have an impact in human mobility behaviour. We complement the model by adding a factor that tests the risk adaptation hypothesis by means of a time-dependent sigmoid function.

We account for the different mobility behaviour of people across states and day of the week, by including a random effect for the state and a random effect for the day of the week. More information on the data sources and models is provided in “Methods”. Alternative model specifications, including different risk adaptation functional forms, are provided in the SI Section S12.

We select, as a baseline, a model that includes as fixed effects only the NPIs stringency and the death ratio over the state population. We evaluate the model through the well-established Bayesian $R^2$^51^ and the PSIS-LOO information criterion^52^. Table 1 shows that this simple model achieves $R^2 = 0.67$ and PSIS-LOO $= 610.60$ and, as expected, shows that the NPIs stringency correlates negatively with the mobility of people. Interestingly, we also find that the death ratio influences the number of visits to POIs.

Then, we account for local weather conditions that might influence the visits to POIs. The Weather model adds the daily precipitations and maximum temperature to the baseline model. Table 1 shows that these two variables significantly increase the model’s performance ($R^2 = 0.72$, PSIS-LOO $= 747.44$) that grow by 7.46% and 22.41%, respectively.

Table 1 shows that the inclusion of the risk adaptation factor (see Full model) achieve the highest performance ($R^2 = 0.78$, PSIS-LOO $= 846.76$). We observe that in this model, the risk adaptation factor represents the second most important factor for understanding the daily visits to POIs. This result holds even when we model the time spent outside the home and when we hypothesise that the risk adaptation depends on the cumulative NPIs stringency, which varies from state to state (see SI Section S12).

**Table 1:** Quantitative results of the Bayesian multivariate linear mixed model to explain the daily number of visits to POIs.

	Baseline	Weather	Full
NPIs stringency	$-0.148 \pm 0.004$	$-0.204 \pm 0.004$	$-0.242 \pm 0.009$
Death ratio	$-0.072 \pm 0.004$	$-0.053 \pm 0.003$	$-0.040 \pm 0.007$
Max temperature	–	$0.009 \pm 0.004$	$-0.001 \pm 0.099$
Precipitations	–	$-0.002 \pm 0.003$	$0.001 \pm 0.100$
Risk adaptation	–	–	$0.118 \pm 0.004$
$R^2$	$0.67 \pm 0.02$	$0.72 \pm 0.02$	$\mathbf{0.78 \pm 0.02}$
PSIS-LOO	$610.60 \pm 31.94$	$747.44 \pm 28.78$	$\mathbf{846.76 \pm 29.59}$

We report the mean and 95% confidence intervals of all the $\beta$ coefficients. We report the mean and standard deviation for $R^2$ and PSIS-LOO.

### Protective behaviour in co-location events

The risk adaptation factor can be interpreted as a change in adherence to COVID-19 protective behaviours and policies. However, it is not clear whether this adaptation only affect people’s tendency to visit more venues or if it is also reflected on the physical distancing patterns.

As a proxy to understand how much people engage in physical and social activities, we empirically define a co-location event as when two individuals stop for at least fifteen minutes and are at most 50 m apart from each other. These events include, among others, people going to the same grocery store, residential area and workplace. We note that our definition of co-location is more robust than previous literature^30,31^ since we represent a social contact from stop locations, thus reporting a co-location event between two people when they share the same stationary point.

We aggregate co-location events in four different categories depending on where the possible social contact took place: (i) *Residential*, a co-location event where only one of the two individuals have the stop marked as *Residential* location; (ii) *Workplace*, a co-location event that happened in a venue labeled as a workplace for both the individuals; (iii) *POI*, a co-location event where both the individuals are in the same POI; and (iv) *Other*, a co-location event in which the two individuals meet in a place that it is neither a *Residential* nor a *Workplace* nor a *POI* (see SI Section S11 for additional details). We here note that *Residential* and *Workplace* co-locations are defined at a coarse level, due to the anonymisation process performed by Cuebiq.

**Figure 5:**
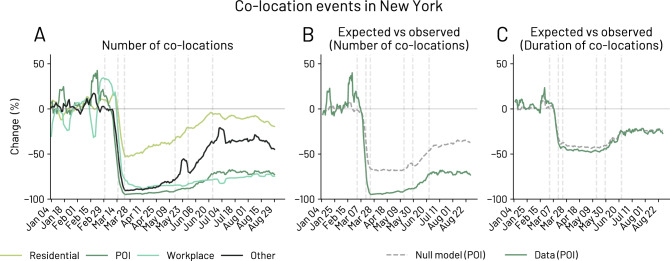
Co-location events. (**A**) Percentage change of co-location events from the baseline (until 29 February 2020) in New York state. We measure the change in co-location events for Residential areas (only one of the two individuals is in proximity of their residential area), POI (both individuals are at the same POI), Workplace (both individuals have the same work location) and Other co-location events. (**B**, **C**) We compare the difference between the expected and observed number and duration of co-location events. During the pandemic, individuals tend to have fewer and shorter co-locations than expected.

Figure 5A shows the abrupt change of the co-location events in the New York state, starting at the school closure day on 15 March and reaching low points of $-95\%$, $-88\%$ and $-90\%$ for *POI*, *Workplace* and *Other* co-location events respectively. Interestingly, we observe that *Residential* co-location events, namely between people who do not live together, experience the smallest reduction, decreasing at most by $53\%$ from the pre-pandemic levels. During the strictest measures put in place in New York state, we notice that people maintain their co-location events inside other’s people residential areas and in places which are not marked as POIs (see *Residential* and *Other* in Fig. 5A). This presumably happens because of the impossibility of having co-location events in venues such as pubs and restaurants (see *Food* and *Nightlife Spot* categories in SI Section S11-A) due to the NPI interventions including physical distancing measures and closures of POIs such as *Arts & Entertainment* and *College & University*.

We obtain similar results in the other states (see SI Section S11), although with some differences. For example, in Oklahoma, Kentucky and Arizona, the *Residential* co-locations events experience a lower reduction, with a low point around $-33\%$ in Arizona and around $-40\%$ in both Oklahoma and Kentucky. Notably, we observe a decrease in co-locations events from the partial reversal of the reopening from 29 June 2020 in Arizona.

Again, we find some differences between the number and the duration of co-location events. First, the duration decrease less than the number of co-location events, reaching low points of around $-10\%$, $-48\%$, $-10\%$, $-30\%$ for *Residential*, *POI*, *Workplace* and *Other* co-location events respectively (see SI Section S11-B).

Overall, we find a strong negative Spearman correlation between the daily number of co-location events and the NPIs stringency in each state ($-0.83$ with p-value $p < 0.001$). Similarly, we find that the daily number of co-location events is also negatively correlated with the daily number of new cases and deceased ($-0.58$ and $-0.67$ respectively, with p-values $p < 0.001$).

The co-location events and the visits to places are inherently connected. Therefore, as soon as the number and the duration of visits decrease, it becomes less probable to have co-location events. Thus, we estimate the daily expected number of co-location events through a null model and compare it with the observed co-locations. The number of co-location events $e_{i,d}$ occurring at a POI *i* on a specific day *d* can be estimated from both the number of individuals visiting the POI $n_{i,d}$, and the median duration of their stops there $\bar{d}_{i,d}$. We can then have an estimate of the co-location events following $$e_{i,d} = \left( {\begin{array}{c}n_{i,d}\\ 2\end{array}}\right) p_{i,d}$$, where $p_{i,d}$ is the probability of having a co-location event given two individuals visiting POI *i* on day *d* and $p_{i,d}$ is computed assuming a uniform distribution for the time-interval of visit of two individuals potentially having a co-location event (see SI Section S11-C for additional details). We follow a similar reasoning to model the expected duration of the individual’s co-location events.

Figure 5B shows that, during the pandemic, the observed number of co-location events at POIs are lower than expected. Similarly, Fig. 5C shows that the duration of co-location events is slightly lower than expected.

We investigate the deviation between the expected and observed number of co-location events and find a high correlation with the daily new cases and deaths (0.66 $p < 0.001$ and 0.48 $p < 0.001$, respectively), and a lower correlation with the NPIs stringency (0.28 $p < 0.001$). Thus, individuals’ discrepancy between the theoretical and observed number of co-location events appear to be largely driven by the epidemic burden (i.e. daily new cases and deaths).

## Discussion

This paper investigates the changes in human mobility behaviour induced by the COVID-19 pandemic in four US states. We exploit a privacy-enhanced longitudinal GPS dataset, counting more than 837,000 anonymous opted-in individuals, to show how individuals changed their patterns of visits, their routinary behaviour and their person-to-person contact activity over time.

We show that, in line with precious research^18,26,32^, the COVID-19 pandemic dramatically reduced the number of visits to POIs, which only partially recovered the pre-pandemic levels ($-28\%$ in the state of New York at the end of the period of study). Additionally, while the duration of visits to POIs decreases less than the number of visits ($-23\%$ at the end of the period), its recovery after the reopening phases is slower. This finding suggests that people are less willing to spend time in POIs, reasonably to minimise social contacts in public venues. This result is strengthened by the analysis of co-location events used as a proxy of social contacts. Since the number of co-location events depends on the number and duration of visits to places, we expect that it becomes less probable to have social contact as soon as these quantities decrease. Nevertheless, we find that individuals have fewer and shorter contacts even after the reopening phases. Despite increasing the number of visits to POIs, individuals display persistent protective behaviours, limiting unnecessary social contacts.

We also find that changes in co-location events are often place-dependent. For example, on average, co-locations events at other’s people residential areas, namely between people who do not live together, reduced by 42%, while co-locations in POIs and workplaces reduce on average by 94% and 73% after the stay-at-home order. While individuals limit unnecessary social contacts, this behaviour shows a limited impact on in-house gatherings. Here, our results reveal a potentially unsafe behaviour, as the literature reports in-house contacts among the ones with a higher risk of infection^53,54^, highlighting the importance of considering *all* co-location places when modelling optimal intervention strategies.

Overall, human routines during the COVID-19 pandemic got shorter and more predictable. The statistical patterns that characterise these new routines (e.g., distribution of travel distance, typical predictability of an individual’s whereabouts) may differ significantly from those observed ubiquitously for pre-pandemic mobility^55,56,56–65^. Further research is needed to understand these differences.

Finally, taking into account the stringency of the NPIs, the local severity of the pandemic, local weather conditions, and a risk adaptation factor, people adapt to the pandemic risk over time, showing an increased tendency in visiting POIs and spending time outside their home.

Multiple reasons may explain the risk adaptation effect. One hypothesis is that risk adaptation arises from a change in the risk assessment of individuals. As the pandemic unfolds, people become less influenced by the number of cases and deaths, therefore paying less attention to protective behaviours. A similar hypothesis sees the risk adaptation as a consequence of the psychological burden, which reduces the ability or motivation to perform self-protective behaviour^66,67^. Another hypothesis considers the sustained economic burden as an important factor, which may force people to decrease policy adherence to go back to work. In all cases, our results well align with previous self-reported evidence^49^ and warn that adherence should not be dismissed and ignored. Long and sustained restrictions to human activities might build up the so-called pandemic fatigue and make NPIs less effective^49^.

Analysing everyday activities from GPS data does not come without limitations. First, the analysis from smartphone data might be biased towards younger adults and fail to capture the mobility of those who do not carry their phones while visiting places. Second, our *Residential* and *Workplace* are just an estimate of the actual home and work locations, which are obfuscated for privacy reasons by the data provider. Third, we acknowledge that our co-location events are a loose proxy of social interactions, and people might share the same location even without knowing each other (i.e., familiar strangers^68^).

COVID-19 disrupted the lives of millions of people. Our work shows that the pandemic-induced changes are not just about how much people stay at home and visit POIs. Instead, the COVID-19 risk and policy interventions reshaped people’s routines and habits, changing how individuals experience POIs, places and social interactions during the pandemic.

Future work might clarify how people adapt to the epidemic risk and the medium- and long-term effects of the COVID-19 pandemic and restriction measures on human behaviour.

## Methods

### Selection of the important dates

We identify several important dates that help readers with the interpretation of our results. In the state of New York, on March 1, the first positive cases were broadly discussed by public opinion. On March 15, Governor Andrew Cuomo announced that New York City schools would have closed from the following day^69^. On March 22, Andrew Cuomo announced the statewide stay-at-home order, also known as the NYS on Pause Program, with a mandate that all non-essential workers work from home beginning at 8 p.m. and that only businesses declared as essential were allowed to remain open^70,71^.

On May 15, Andrew Cuomo announced a gradual plan of reopening, also called Phase I. From May 28, the New York State Department of Health released the guidelines for the reopenings of Phase II, which included the reopening of professional services including finance and insurance, retail, administrative support, and real estate/rental leasing^72^. We note that New York City met the criteria on June 22. On June 15, Andrew Cuomo announced that regions upon entry of Phase 3 will be allowed non-essential gatherings of up to 25 people and that on-location restaurants would open^73^. New York City entered this phase on July 6. We highlight some of these dates in the plots, and we describe the important days for all the states in SI Section S5-A.

### Stop location detection

We use GPS location data provided by Cuebiq, a location intelligence company that shared a dataset consisting of anonymised GPS locations from users that opted-in to share the data anonymously for research purposes through a CCPA (California Consumer Privacy Act) compliant framework. To further preserve privacy, the data provider obfuscates users’ precise home and work locations by transforming it to the centroid of the corresponding Census Block Group.

The dataset span a period of 9 months, from January 2020 to September 2020 (details in SI Section S2). The data is provided through the Cuebiq Data for Good COVID-19 Collaborative program, which provides access to de-identified and privacy-enhanced mobility data for academic research and humanitarian initiatives only.

To ensure the data describes people’s mobility throughout the pandemic, we filter out all users with less than one month of data before declaring a national emergency (March 13, 2020) and less than four months after it. We also require users have 5 hours per day covered by at least one GPS location. The resulting dataset includes more than 837,000 anonymous, opted-in individuals.

For all users, we extract their stop events with an algorithm based on Hariharan and Toyama^37^. A stop event is defined as a temporal sequence of GPS coordinates in a radius of $\Delta _s$ meters where a user stayed for at least $\Delta _t$ minutes. The algorithm, its optimisation, and its computational complexity are explained in detail in SI Section S3. To define a stop event, we used $\Delta _s=65$ meters and $\Delta _t = 5$ minutes due to the distribution of accuracy of the underlying data (see SI Section S3).

For each user, we then define their stop locations as the sequences of stop events that can be considered as part of the same place. To determine a stop location from a sequence of stop events we use the DBSCAN algorithm^38^. With DBSCAN, we group points within a distance of $\varepsilon = \Delta _s - 5$ meters to form a cluster with at least $\text {minPoints}=1$ stop event (see SI Section S3 for more details).

### Point of Interest (POIs) association

We extract all POIs from OpenStreetMap (OSM) (https://www.openstreetmap.org/) and then, due to the lack of structure in OSM POIs, we map each OSM POI to the corresponding Foursquare Venue Category Hierarchy (https://developer.foursquare.com/docs/build-with-foursquare/categories/). After the association, each OSM POI is mapped to the Foursquare categorisation with 8 first-level categories and 178 second-level categories. Further details are described in the SI Section S7.

Then, we associate each stop location to its nearest POI for all users whenever the Haversine distance is less or equal to 65 meters. Since in OpenStreetMap POIs can be represented as Points or Polygons, sometimes nested, we assign POIs to stop locations with the heuristic described in SI Section S7.

### Percentage change

Throughout the analysis we compute the change with the same methodology of the Google mobility reports^26^. Specifically, we compute the percentage change as:

$$\begin{aligned} P_{i} = \frac{v_{i} - b_{w(i)}}{b_{w(i)}} * 100 \end{aligned}$$

where $v_i$ is the original value at day *i* and $b_{w}$ is the median value at day of the week *w*(*i*), going from 0 to 6, computed during the baseline period (i.e. before the pandemic).

### Sequitur, significant routine patterns, and user-user similarity

Sequitur is a compression algorithm that reduces a sequence size by introducing new symbols/words when in the original sequence appear repetitions of short sub-sequences and motifs^45^. We represent an individual’s mobility through a sequence of symbols that maps a stop location into a category (e.g., *Residential*, *Workplace*, *Food*). From these sequences of symbols we extracted recurrent patterns of visits consisting of sub-sequences of length $l\ge 2$ following Di Clemente et al.^44^.

We focus our attention on two 4-week periods. The first one starts on February 1, 2020 and excludes January, which might display unusual patterns due to seasonal effects (e.g., the end of the holiday period). The second one starts at the beginning of each state emergency response orders, i.e. the “stay-at-home” order (in Kentucky, we selected the “healthy-at-home” order since no “stay-at-home” was ever issued). This period terminates before the first reopenings to include the most stringent early regulatory phase for each state (for more details on relevant dates see SI Table S1).

For each individual sequence of visits, we randomly shuffle the sequence 1000 times and apply the sequitur algorithm on each one of them^45^. For each symbol *s* (a visit in our case), we compute the mean number of occurrences, $\mu _s$ and its standard deviation, $\sigma _s$, across all random synthetic sequences. We use this quantity to compute the standard score, $z_s=\frac{o_s-\mu _s}{\sigma _s}$, where $o_s$ is the number of occurrences of each symbol *s* within the original sequences. Significant routines are selected from all the sub-sequences if $z_s$ is greater than 2. We refer to significantly recurrent sub-sequences as “routines”.

### Modeling expected number and duration of co-location events

The expected number of co-location events is assumed to depend on both the number of individuals visiting a POI, *i* on day *d*, and the average duration of their stop there, $d_{i,d}$. The first quantity is used to compute the combinations of possible co-location between different individuals. The second is used to estimate the probability $p_{i,d}$ of having two temporal interval of duration $\bar{d}_{i,d}$ overlapping by at least 15 minutes over the entire time span of a day. Combined together, our estimate of the number of co-location events, $e_{i,d}$, can be written as: $$e_{i,d} = \left( {\begin{array}{c}n_{i,d}\\ 2\end{array}}\right) p_{i,d}$$.

Following similar reasoning, the average duration of co-location events is estimated conditioning on the occurrence of the event and assuming that the temporal overlap of two individuals, $o_{i,d}$, depends on their average permanence at POI *i*, $\hat{d}_{i,d}$. Thus, the average overlap area can be straightforwardly computed as: $o_{i,d} = \frac{\hat{d}_{i,d}-15}{2}$. More information about the formulation and computations in support of these models can be found in SI Section S11-C.

### Modeling visits to Points of Interest

We model the daily average number of visits to POIs *Y* with a Bayesian linear regression formulated for each day of year *d*, state *s* and weekday *w* as:

1 $$\begin{aligned} \begin{aligned} Y_{d, s, w}&\simeq \alpha _s + \beta _{\text {policy}} S_d + \beta _{\text {deaths}} D_{d-1} + \beta _{\text {temp}} T_d + \\ \beta _{\text {prec}} P_d&+ \frac{\beta _{\text {adapt}}}{1+e^{-\gamma _s (d-\phi _s)}} + \rho _w + \varepsilon , \end{aligned} \end{aligned}$$

where $\varepsilon$ is the residual error, $\alpha _s$ is the state-specific intercept, $\beta _{\text {policy}}$, $\beta _{\text {deaths}}$, $\beta _{\text {temp}}$, $\beta _{\text {prec}}$ and $\beta _{\text {adapt}}$ are the $\beta$ coefficients for each independent variable in the regression, while $\rho _w$ is the week-day random effect controlled by the variable *w*, the day of the week of (from 0 to 6 where 5 is Saturday and 6 is Sunday). $S_d$ is the value of the Stringency Index, measuring local enacted regulations and preventive and informative campaigns (see^74^ for more details), $D_{d-1}$ is the death ratio at the previous day (measured over a population of 100k people), $T_d$ is the maximum temperature in Celsius degrees and $P_d$ represents the millimeters of precipitations. $T_d$ and $P_d$ account for the seasonal effects of POI visits. Then, $\frac{1}{1+e^{-\gamma _s (d-\phi _s)}}$ is a standard sigmoid function that models the collective behavioural adaptation of people to the perceived epidemic risk. The sigmoid function depends on time where $\phi _s$ and $\gamma _s$ model the location and the sharpness of the sigmoid, respectively. All the independent variables are z-score standardised.

We extract the daily temperature and precipitation from the PRISM Climate Group^75^, which provides the maximum temperature and precipitations with a 4km grid. Then, we compute the average maximum temperature and precipitations for each state. Finally, the average is weighted with the population of each county to account for the number of people exposed to the measured temperature and precipitations.

We assess the out of sample predictive accuracy through the Pareto-smoothed importance sampling Leave-One-Out cross-validation (PSIS-LOO)^52^. This metric overcome the issues of the Deviance Information Criterion (DIC)^76^ such as its lack of consistency and the fact that is not a proper predictive criterion^52,77^, and it has rapidly become state of the art for evaluating Bayesian models. The PSIS-LOO is defined in the log score as:

2 $$\begin{aligned} \text {PSIS-LOO} = \sum _{i=1}^n \ln \left( \frac{\sum _{s=1}^S w_i^s p(y_i|\theta ^s)}{\sum _{s=1}^S w_i^s}\right) . \end{aligned}$$

where *n* is the number of data points, $\theta ^s$ are draws from the full posterior $p(\theta |y)$, $s=1,\dots ,S$ represent the *S* draws, and $w_i^s$ is a vector of weights that are the Pareto Smoothed importance ratios built through an algorithm described in the PSIS-LOO original paper^52^. The best model is associated with the highest PSIS-LOO value. We also report Bayesian $R^2$^51^ as an additional and easy-to-interpret measure of goodness of fit.

## Supplementary Information

Supplementary Information.

## Data Availability

Replication code is available on GitHub at https://github.com/denadai2/living-the-pandemic. All the data sources are freely available on the Internet while the mobility data from Cuebiq can be accessed only through the Data for Good initiative of the company (https://www.cuebiq.com/about/data-for-good/). Limitations apply to the availability of this data, due to the rigorous anonymity constraints.
