# Supplementary material for: Living in a pandemic: changes in mobility routines, social activity and adherence to COVID-19 protective measures
Source: Sci Rep. 2021 Dec 27;11:24452. doi: 10.1038/s41598-021-04139-1 (PMC8712525; doi:10.1038/s41598-021-04139-1)
Supplement: Supplementary file 1 — Supplementary Information. [file 41598_2021_4139_MOESM1_ESM.pdf]

Supplementary Information of: Living in a pandemic: changes in mobility routines, social activity and adherence to COVID-19 protective measures

Lorenzo Lucchini,[1,*] Simone Centellegher,[1,*] Luca Pappalardo,[2] Riccardo Gallotti,[1] Filippo Privitera,[3] Bruno Lepri,[1] and Marco De Nadai[1]

[1]Fondazione Bruno Kessler (FBK), Trento, Italy

[2]Institute of Information Science and Technologies, National Research Council (ISTI-CNR), Pisa, Italy

[3]Cuebiq Inc., New York, NY, USA

Contents

S1. New York City before and during the pandemic 3

S2. Datasets 4

A. Location data 4

B. COVID-19 government response measures 5

S3. Stops detection 5

S4. Residential/Workplace detection 8

S5. States selection 9

A. Important dates 10

S6. Users selection 12

S7. Points of interest 13

A. Extraction of Points of interest (POIs) 13

B. Popularity of POIs in the US 13

C. Matching stop locations with points of interest 16

D. New York state - Number and duration of visits to POIs 19

E. Change in POIs' visits in Arizona, Kentucky and Oklahoma 22

*These two authors contributed equally.

Correspondence to: lorenzo.f.lucchini.work@gmail.com and work@marcodena.it.

F. Essential vs non-essential Shop & Service 25

S8. Comparison with Google and Foursquare mobility reports 26

S9. Behaviour change metrics 26

A. Entropy of visited locations 28

B. Radius of gyration 28

S10. Mobility routines 28

A. Comments on significant routines 29

B. Sequitur compression ratio 30

C. Routine reduction 34

D. Jaccard similarity and clustering 34

E. Top clusters 35

S11. Co-location events 36

A. Percent change of the number of Co-location events 37

B. Percent change of the duration of Co-location events 40

C. Null model 43

S12. Visits to POIs: model construction and model selection 44

References 48

S1. NEW YORK CITY BEFORE AND DURING THE PANDEMIC

Figure S1 shows more than 9 million stop locations to POIs in New York City before and after the stay-at-home order. On average, we observe a 60.6% reduction of visits to POIs in the state of New York, with reduction of −87.7%, −83.7%, and −77.7% for *College & University*, *Nightlife Spot*, and *Arts & Entertainment*, respectively. As a concrete example, Figure S1 highlights the number of stop locations at the Metropolitan Museum of Art, one of the most popular *Arts & Entertainment* venues in New York City; during the pandemic, it almost disappears from the map.

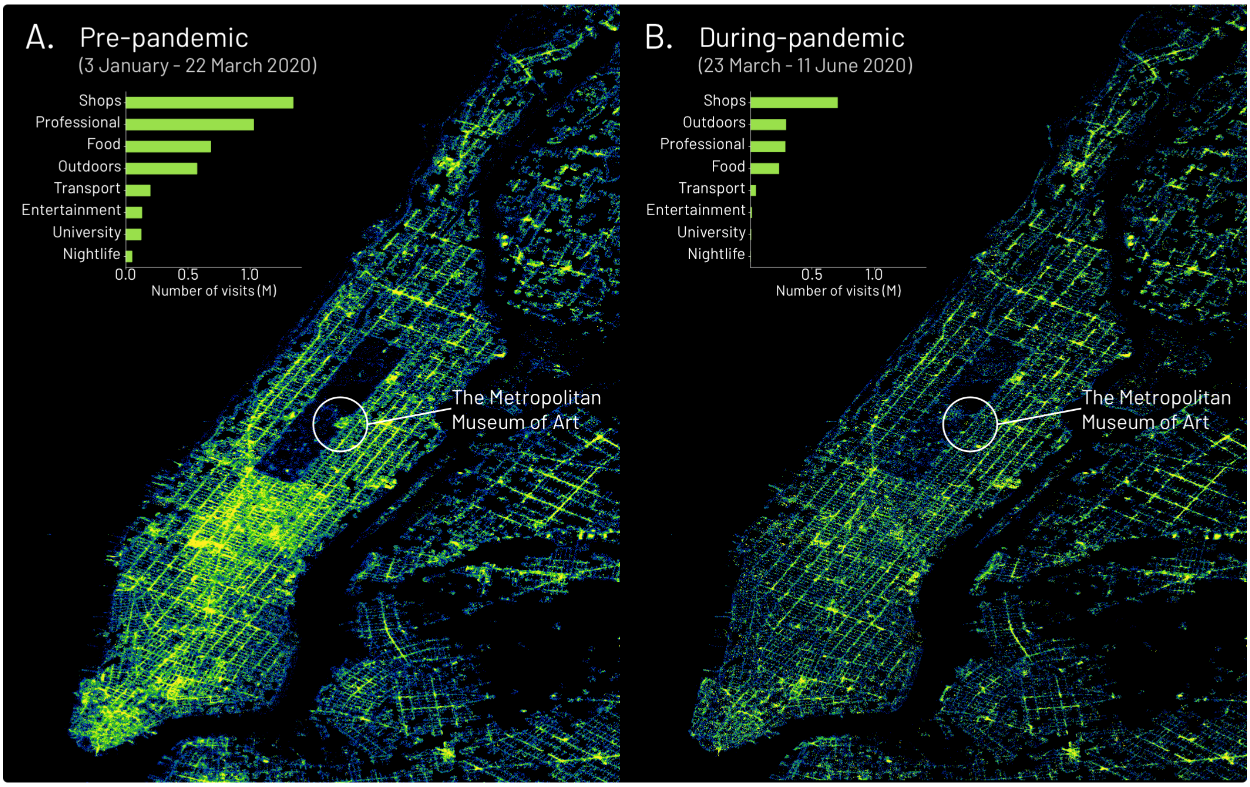

FIG. S1: (A) Number of visits to POIs in New York City in the 82 days before the stay-at-home order (22 March 2020) and (B) the 82 days after the stay-at-home order. In the state of New York, we find a decrease of 60.6% of the visits to the POIs, revealing the dramatic change in visiting habits brought about by the pandemic in 2020. In the insets, we show the distribution of the visits to the POI categories in the state of New York. As a concrete example, we highlight how activity at the Metropolitan Museum of Art in New York City experiences a drastic reduction of visits during the pandemic. The figure was produced with Datashader.

S2. DATASETS

A. Location data

The location data is provided by Cuebiq Inc., a location intelligence and measurement company. The dataset was shared within the Cuebiq Data for Good program, which provides access to de-identified and anonymized mobility data for academic and research purposes.

The location data used consists of users in the US over nine months, from January 2020 to August 2020, and includes only users who have opted-in to share their data anonymously. The data is General Data Protection Regulation (GDPR) and California Consumer Privacy Act (CCPA) compliant. Furthermore, to increase and preserve users' privacy, Cuebiq obfuscates home and work locations to the census block group level.

The data is collected through the Cuebiq Software Development Kit (SDK) that collects user locations through GPS and Wi-Fi signals in Android and iOS devices.

The device determines the location accuracy, which varies from 0 to more than 100 meters. Figure S2 (left) shows the accuracy in meters of the original GPS events in the dataset. We can see that the accuracy distribution is bimodal, with one peak around 5 meters and the other peak at 65 meters. We speculate that the latter peak is caused by the home obfuscation mechanism of Cuebiq to preserve users' privacy. Figure S2 (right) shows the average number of hours per day with at least one GPS location per user. We can see that most users have almost all the hours covered, enabling us to describe human behaviour accurately.

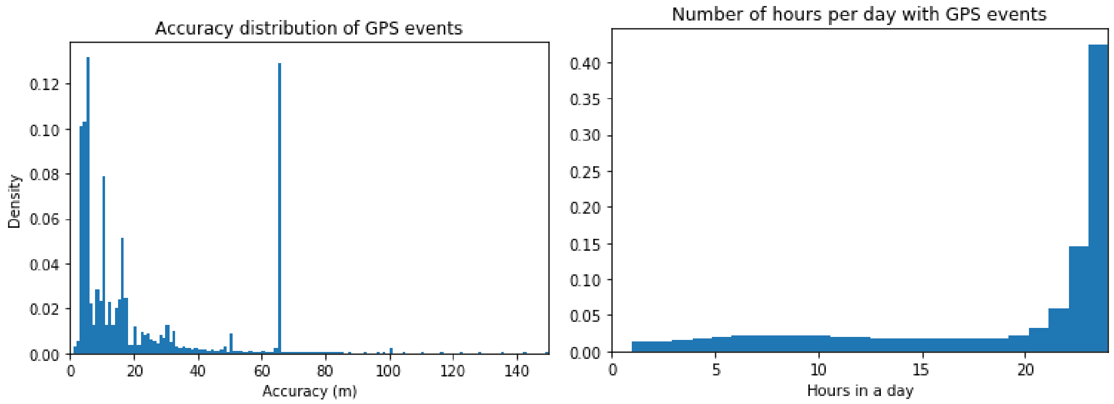

FIG. S2: (left) Accuracy distribution of Cuebiq GPS events. (right) Distribution of the average number of hours (per user) covered by at least one GPS event.

B. COVID-19 government response measures

During the COVID-19 pandemic, governments have put in place non-pharmaceutical interventions (NPIs) to contain the spread of the virus. These measures include, among others, school closures, travel and movement restrictions, bans on public gatherings. We used the dataset provided by the Oxford COVID-19 Government Response Tracker (OxCGRT), which provides a systematic set of measures of governments' policies and interventions from 1 January 2020, providing a standardized series of indicators. The dataset covers over 180 countries, including sub-national measures enacted in the United States. The state-specific information included in the dataset describes the overall policy stringency under which residents of the state are subjected. The US states policies are quantified using indicators inherited from higher-level governments and their policies. State-level policies are built from regulations made at the state level and below (e.g., indexes also include decisions of county or city governments) [11]. As an aggregate measure of the strength of the enacted regulations, we use the "Stringency Index" [7]. The index is composed of nine different indicators, each quantifying the strength of the enacted policies on a specific regulatory category both concerning the containment and closure policies and the health care system policies. Here we report the nine indicators (and their respective range of values) from which the "Stringency Index" is constructed (for a more detailed description on how these indexes are aggregated, see [7] or [11]):

- school closure (from 0 to 3);
- workplace closure (from 0 to 3);
- cancel public events (from 0 to 2);
- restrictions on gatherings (from 0 to 4);
- public transport closure (from 0 to 2);
- stays at home requirements (from 0 to 3);
- restriction of internal movements (from 0 to 2);
- restriction on international travels (from 0 to 4);
- intensity of public information campaigns (from 0 to 3).

S3. STOPS DETECTION

A single original GPS location does not contain any information about people's movements, and it is thus impossible to know, from a single point, whenever a user is stationary in a place or not.

Moreover, the coordinates of GPS points can fluctuate over time even when the user is not moving. Therefore, we apply a stop location detection algorithm to transform a sequence of original GPS data into a sequence of locations in which a user stops.

We detect the users' stops in a two-step algorithm composed of detecting (i) stop events and (ii) stop locations.

Stop events detection. From a sequence of ordered time events $T = [t_0, t_1, \ldots, t_n] \mid t_j \leq t_i$, a corresponding set of GPS locations $R = [r_0, r_1, \ldots, r_n]$, and a geographical distance function $d(i, j)$, we define a *stop event* as a maximal set of locations $S = [r_i, r_{i+1}, \ldots, r_j] \mid d(r_i, r_j) < \Delta s \wedge t_j - t_i \geq \Delta t \; \forall r_i, r_j \in S$.

Then $\mathcal{S} = \{S_i \mid S_i \text{ is a stop event} \wedge r_i \in S_i \wedge r_j \in S_j \wedge i < j\}$ represents the set of *stop events*. To form a *stop event* we heuristically choose to group locations in a time-ordered fashion. In other words, in this step, we aim at finding all those places at most Δs meters large where people stopped for at least Δt minutes. Each *stop event* is composed of at least two locations, and the locations can belong only to at most one *stop event*.

To extract *stop events* we base our method on Hariharan and Toyama's work [8]. The algorithm is depicted in Algorithm 1 and can be summarised as follows: for each user, we first order their GPS locations by timestamp, and then we select groups of GPS sequences with the desired spatial (Δs) and temporal (Δt) thresholds to form *stop events*.

The `Diameter` function computes the greatest distance between points, while the `Medoid` function selects the GPS location with the minimum distance to all other points in the set.

The computational complexity of the *stop event* algorithm [8] is $\mathcal{O}(n^3)$, because of the repeated `Diameter` function that computes a distance matrix, whose complexity is $\mathcal{O}(n^2)$.

To reduce the computational burden of the algorithm, we divide the sequence of points of each user into buckets/chunks. To do so, we use the algorithm we depict in Algorithm 2. Then, we horizontally parallelize the `Diameter` algorithm across users and chunks, as each chunk can compute the stop locations independently from the others.

The `Diameter` $d(i, j)$ is computed through the Haversine great-circle distance between r_i and r_j. Given the average radius of the Earth r and two points with latitude and longitude φ_1, φ_2 and λ_1, λ_2 respectively, the Haversine distance d between them is:

$$d(r_1, r_2) = 2r \arcsin\left(\sqrt{\sin^2\left(\frac{\varphi_2 - \varphi_1}{2}\right) + \cos(\varphi_1)\cos(\varphi_2)\sin^2\left(\frac{\lambda_2 - \lambda_1}{2}\right)}\right).$$

GPS locations fluctuate quite significantly, especially when mobile phones are used indoor.

Algorithm 1: Algorithm for extracting the *stop events* from original GPS sequences.

Input: Time-ordered list of a user's original GPS positions $R = [r_0, r_1, \ldots, r_n]$, their timestamp $T = [t_0, t_1, \ldots, t_n]$, a spatial threshold Δs and a temporal threshold Δt.

Output: The set S of a user's stop events.

```
left = 0; S ← ∅ ;
while left < n do
    right ← minimum j such that t_j ≥ t_left + Δt;
    if Diameter(R, left, j) > Δs;
     then
        left ← left +1;
    end
    else
        right ← maximum j such that j ≤ n and Diameter(R, left, j) < Δs;
        S ← S∪ (Medoid(R, left, right), t_left, t_right) ;
        left ← right +1;
    end
end
```

Algorithm 2: Algorithm for dividing an user's *stop events* into buckets.

Input: Time-ordered list of a user's original GPS positions $R = [r_0, r_1, \ldots, r_n]$ and the spatial threshold Δs.

Output: The set B buckets of an user's stop events.

```
i = 0; j = -1; while i < n do
    if i = 0 or d(r_{i-1}, r_i) > Δs;
     then
        j ← j + 1;
        B_j ← ∅;
    end
    B_j ← B_j ∪ {r_i} ;
    i ← i + 1;
end
```

Thus, we measure how much the GPS locations fluctuate within a *stop event*. For each *stop event*, we compute the average Haversine distance of each GPS location from the medoid (Figure S3).

Stop location detection. For each user, we define *stop locations* as the sequences of *stop events*

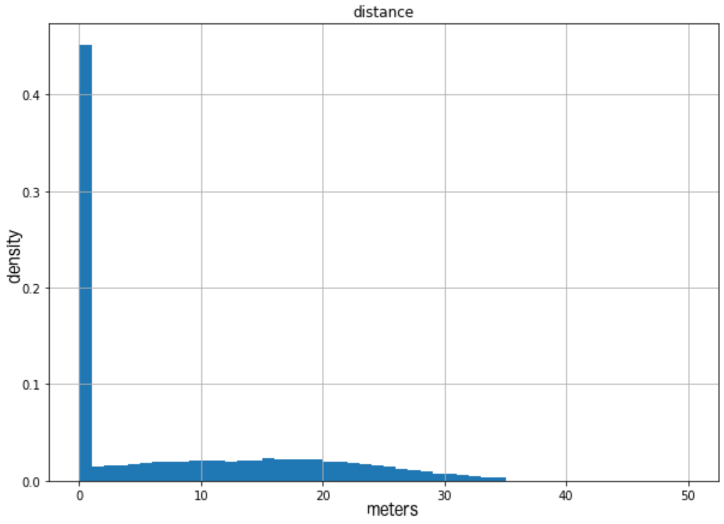

FIG. S3: Distribution of distances between GPS points within a *stop event* and the medoid chosen as representative point for the *stop event*

that can be considered part of the same place. For example: if user U goes to the Colosseum in Rome multiple times, U could have multiple *stop events* (e.g., northern entrance, southern entrance) that can be grouped in a unique *stop location* (i.e. the Colosseum).

To detect a *stop location* from *stop events* we use the DBSCAN [5] algorithm that groups points within $\epsilon = \Delta s - 5$ meters of distance to form a cluster with at least $minPoints = 1$ *event*. The complexity of DBSCAN is $\mathcal{O}(n)$. We horizontally scale the computation through different cloud machines thanks to Apache Spark.

Following the distribution of accuracy in Figure S2, we choose $\Delta s = 65$ meters. We also choose $\Delta t = 5$ minutes to detect all stops from the shortest to the longest ones.

We select $\epsilon = \Delta s - 5$ meters to avoid the creation of an extremely –and incorrect– long chain of sequential *stop events*.

S4. RESIDENTIAL/WORKPLACE DETECTION

To analyze human mobility in detail, we need to classify each user's stop location semantically. For this reason, we computed the most probable residential and workplace areas for each user. Furthermore, to capture possible changes in residential and workplace areas due to the pandemic, we computed these areas multiple times for a moving window of 28 days. We proceed as follow, for each user i and stop $s_i(t)$ in a day t, we aggregate the time spent in $[t - 14, t + 14]$ days into two buckets:

1. **Potential residential time.** We sum the time between 8 pm and 4 am spent in stop s_i; differently from previous literature (e.g., TimeGeo [9]), we do not assume that the entire weekend is potential residential time as the US Bureau of Labor Statistics recently estimated that between 34% of employed people work in the weekend [2].

2. **Potential work time.** We sum the weekday time between 9 am and 5 pm spent in stop s_i. Moreover, we assume that a potential work stay should last at least 30 minutes and have a frequency of 5 visits per week. These assumptions are similar to previous literature [9]. We choose the aforementioned working hours since they represent the most popular working time in the US [15].

For each day t, we label a stop $s_i(t)$ as *Residential stop* if this stop has the largest *potential Residential time*. Then, we label a stop $s_i(t)$ as *Work stop* if this stop is not *Residential stop* and it has the largest *potential Work time*.

Limitations. It is challenging to extract home and work locations for night and weekend jobs. The former have work times complementary to the residential times of daily jobs. The latter are characterized by few work hours that might make the work detection noisy if considered. The problem is well known by existing literature and, to the best of our knowledge, there are no high-quality datasets of home and work locations to systematically validate existing algorithms. However, we did not find any large non-residential area with a large number of home locations.

S5. STATES SELECTION

We aim at understanding whether there were differences in the mobility behaviour patterns of individuals in states that put in place different strategies in response to the COVID-19 pandemic.

To select these four US states, we took into account (i) the daily COVID death rate and (ii) the stringency measures the states adopted to slow down the spread of the virus. Both information was retrieved from the OxCGRT dataset [7]. As shown in Fig S4 we selected the states of Arizona (many deaths/low stringency), Oklahoma (few deaths/low stringency), Kentucky (few deaths/high stringency) and New York (many deaths/high stringency).

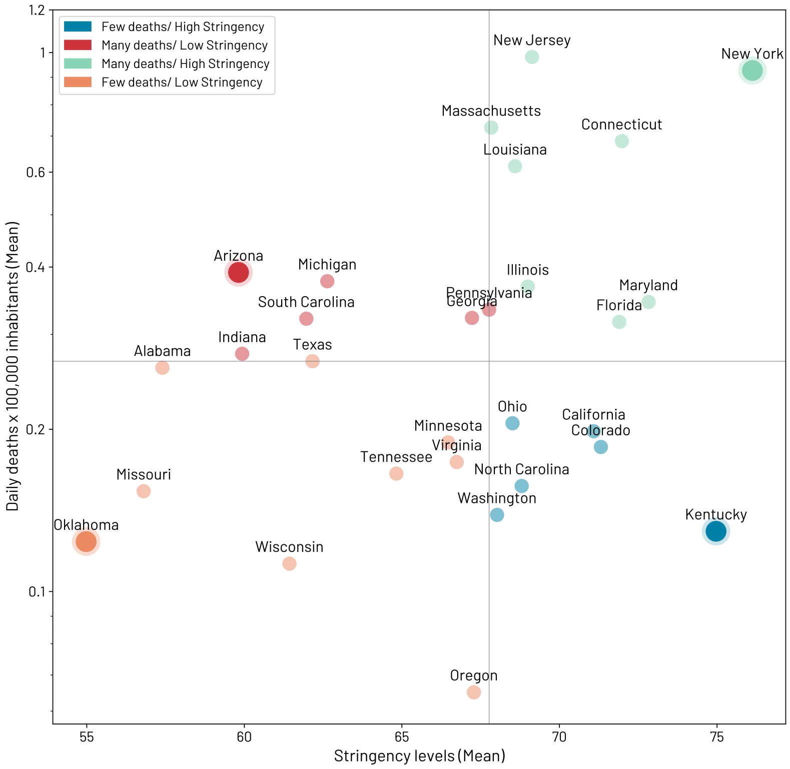

FIG. S4: State selection (AZ, KY, NY, OK) based on daily deaths per 100K inhabitants and mean stringency level for states that have a population > 1% of US total population.

A. Important dates

We retrieved individual state dates for relevant events concerning the pandemic and the local regulatory response from state-dedicated web pages on the Wikipedia website [17]. We here summarize the main events, focusing on the first case reported within the state borders and the main regulatory responses. The dates are conveniently reported also in Table S1.

Arizona

- First case reported: 26 January 2020;
- Universities start to move to online courses: 12 March 2020;
- Governor issues a statewide "stay-at-home" order, barring Arizonans from leaving their houses except for essential activities: 31 March 2020;
- A partial reopening for a selected set of non-essential activities is announced: 4 May 2020;
- The "stay-at-home" order expires: 15 May 2020;
- Following a record high in the number of hospitalizations a partial reversal of the "stay-at-home" order is announced: 29 June 2020.

Kentucky

- First case reported: 6 March 2020;
- School closure: 16 March 2020;
- Governor issues a ban for mass-gatherings: 20 March 2020;
- Non-essential business closure is enacted and a “healthy-at-home” order (similar to a “shelter in place” policy): 26 March 2020;
- Reopening starts requiring business to follow public health guidelines (“healthy-at-work” initiative): 9 May 2020;
- Restaurants reopening: 22 May 2020;
- Bars reopening operating (similarly to restaurants) at a 50% indoor capacity: 29 June 2020.

New York

- First case reported: 1 March 2020;
- School closure in New York city: 15 March 2020;
- Statewide “stay-at-home” order is declared, all non-essential business are ordered to close: 22 March 2020;
- “Phase one”, a county-level partial reopening upon meeting qualifications is announced: 15 May 2020;
- “Phase two”, if meeting qualifications counties can reopen restaurants outdoor activity as in-store activities for specific shop categories: 29 May 2020;
- “Phase three”, if meeting qualifications counties can reopen indoor restaurant activity and bars at 50% capacity: 24 June 2020.

Oklahoma

- First case reported: 7 March 2020;
- Announcement of a “safer-at-home” order requiring vulnerable people to remain at their residences except for essential activities, “school closure” order and ban of mass gatherings are enacted on the following day: 24 March 2020 (and 25 March 2020);
- “Phase one” of business reopening allows for outdoor activities, personal care facilities, restaurants, cinemas, gyms, and places of worship to restart activities subject to physical distancing and sanitation protocols (specific guidelines are issued for the industry): 24 April 2020;
- “Phase two” allows religious ceremonies, bars to reopen, and organized sports to restart activities subject to physical distancing: 15 May 2020;
- “Phase three” reopens business that has been restricted to appointments only, summer caps

and (although limited) also visitation at hospitals: 1 June 2020.

	AZ	NY	OK	KY
**first case**	2020-01-26	2020-03-01	2020-03-07	2020-03-06
**stay-at-home**	2020-03-31	2020-03-20	2020-03-24	-
**healthy-at-home**	-	-	-	2020-03-26
**school closure**	-	2020-03-15	-	2020-03-16
**university online**	2020-03-12	-	-	-
**stay-at-home expired**	2020-05-15	-	-	-
**partial reversal stay-at-home**	2020-06-29	-	-	-
**partial reopening**	2020-05-04	-	-	-
**phase-one**	-	2020-05-15	2020-04-24	-
**phase-two**	-	2020-05-29	2020-05-15	-
**phase-three**	-	2020-06-24	2020-06-01	-
**start reopening**	-	-	-	2020-05-09
**restaurants reopening**	-	-	-	2020-05-22
**bars reopening**	-	-	-	2020-06-29

TABLE S1: Dates of the non-pharmaceutical intervention phases for each state.

S6. USERS SELECTION

For each US state we studied, we performed all our analyses on panels of individuals that:

1. have been seen for at least 7 days before the stay-at-home declaration and that have more than 5 hours of activity on average;

2. have been seen for at least 7 days after the stay-at-home declaration and that have more than 5 hours of activity on average.

This pre-processing step leaves us with individuals for which we have information before and after the stay-at-home declaration, enabling a more sound analysis of how mobility behaviour changed during the pandemic.

S7. POINTS OF INTEREST

A. Extraction of Points of interest (POIs)

We downloaded the POIs from OpenStreetMap (OSM) for the entire US. More precisely, we extracted the POIs with the Python package Pyrosm[13], which reads the data from an OSM public dump.

OSM is a collaborative project with maps created by everyone. The nature of the project and its tagging system - which has a quite free form - create some inconsistencies since it allows a user to insert a POI in different ways. As an example, if users want to add a *hospital* in OSM, they can do it setting *healthcare=hospital* or setting *amenity=hospital*.

Another disadvantage of the OSM tagging system is that the POI taxonomy is not organized hierarchically and thus it does not exists a unique way to organize the POIs. To overcome these problems, we decided to create a manual mapping between the OSM tags [16] and the Foursquare Venue Category Hierarchy [1]. In all the analyses in the manuscript, we will therefore use Foursquare categories.

Finally, we retained only POIs that represent actual places where people can meet or go, such as for leisure activities (e.g., parks, sports facilities) or for business reasons (e.g., offices). We decided to remove POIs such as highways, sheds, landuse, fountains, as they do not represent interesting venues. Moreover, we kept only POIs that had a mapping with at least a Foursquare second-level categorization.

B. Popularity of POIs in the US

After the POIs extraction phase, we remain with a total of 3,353,502 POIs for the entire US. In Fig S5 we show the number of OSM POIs mapped to Level 1 Foursquare categories that we extracted for the four states analyzed. In the same way, Fig S6 shows the number of OSM POIs mapped to Level 2 Foursquare categories.

As shown in Fig S5, the majority of POIs extracted from OSM are related to *Outdoors & Recreation* activities as also reflected in the Level 2 Foursquare categories *Park* and *Athletics & Sports* (Fig S6).

The second most common category is *Professional & Other Places* which contains venues such as *Office*, *Factory* and *School* (e.g., Elementary School, High School, Music School, etc.) which appears at the top of Fig S6.

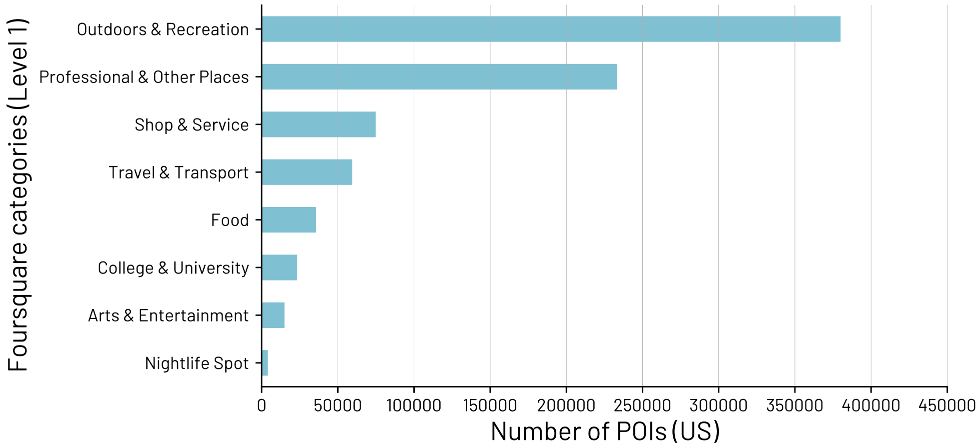

FIG. S5: Level 1 Foursquare categories mapped from Open Street Map POIs downloaded for the four states analysed.

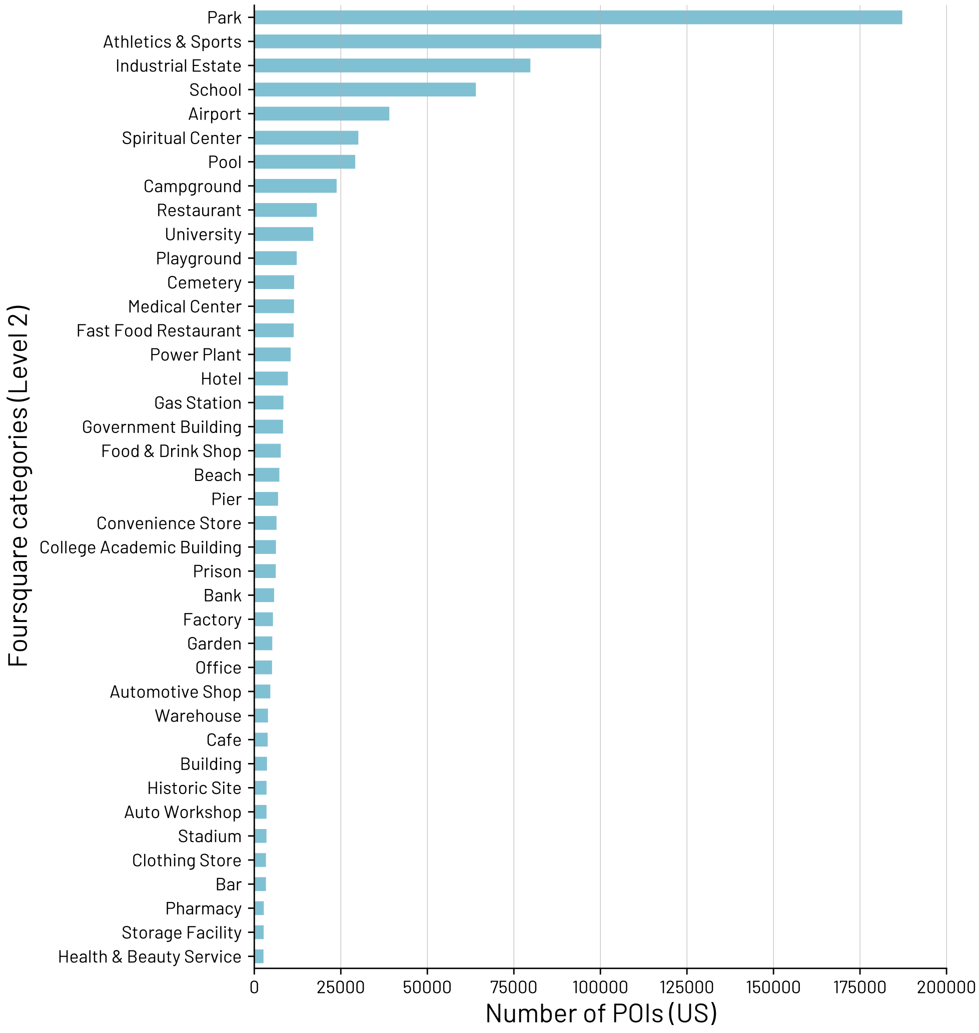

FIG. S6: Top-40 Level 2 Foursquare categories mapped from Open Street Map POIs for the four states analysed.

C. Matching stop locations with points of interest

To further enrich our analysis, we added semantic meaning to individuals' trajectories associating stop locations to their nearest POI when looking at the distance between the stop location and the POI.

When we associate a POI to a stop location, we prioritize a Point POI over a Polygon POI, checking whether a POI is inside another POI. This situation happens, for example, when an individual is inside a shopping mall.

In this case, we will have a POI representing the entire shopping mall (Polygon POI), and the distance between the stop location of the individual and the POI will be 0. Now, suppose that we have a cafeteria (Point POI) inside the shopping mall, which is at 3 meters distance. In this case, we will assign the stop location to the cafeteria since it gives us a fine-grained piece of information on the visit patterns of the individual.

In Fig S7 we show the number of visits made by individuals in the four selected US states (AZ, KY, NY, OK) to POIs mapped at Level 1 Foursquare categories, and in Fig S8 at Level 2 Foursquare categories. Following Cuebiq's policy we removed *health-related* categories, "Spiritual Centers", "Governmental Buildings", and "Prisons" [3].

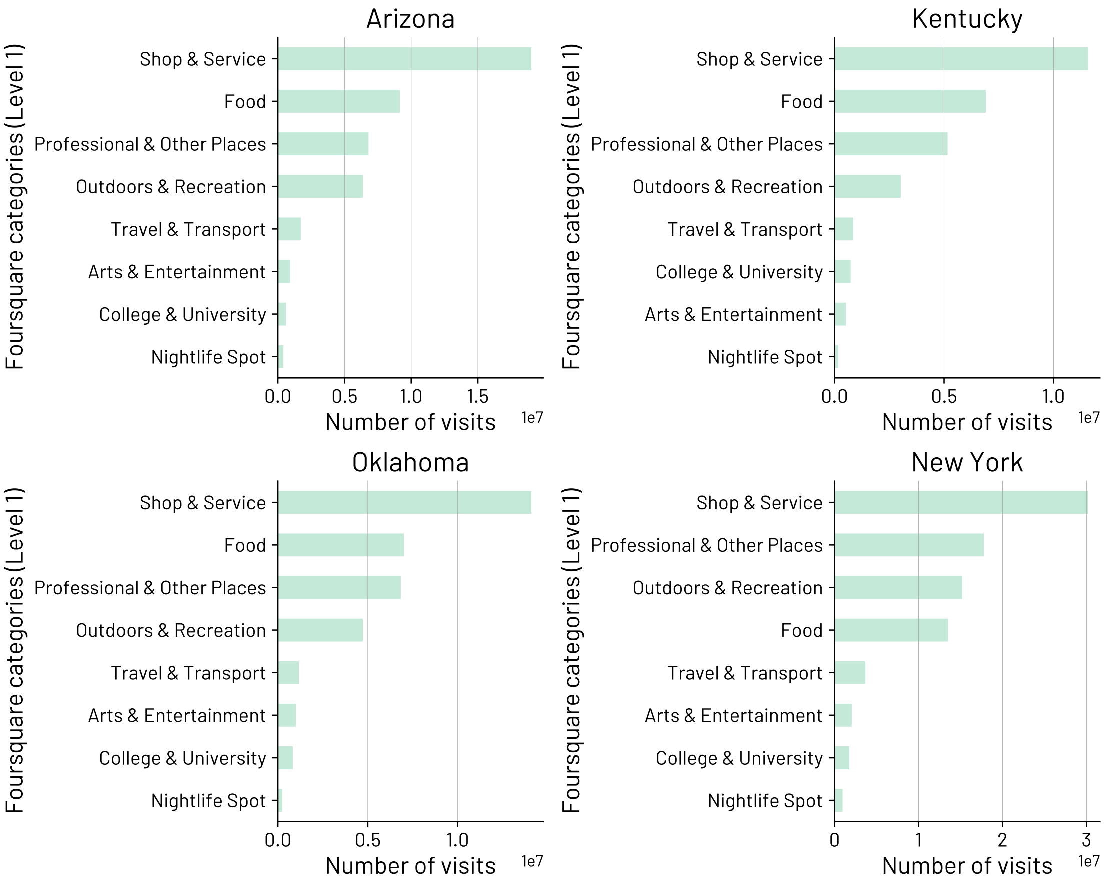

FIG. S7: Number of visits made by individuals in the four selected US states (AZ, KY, NY, OK) to POIs mapped at the Level 1 Foursquare categories

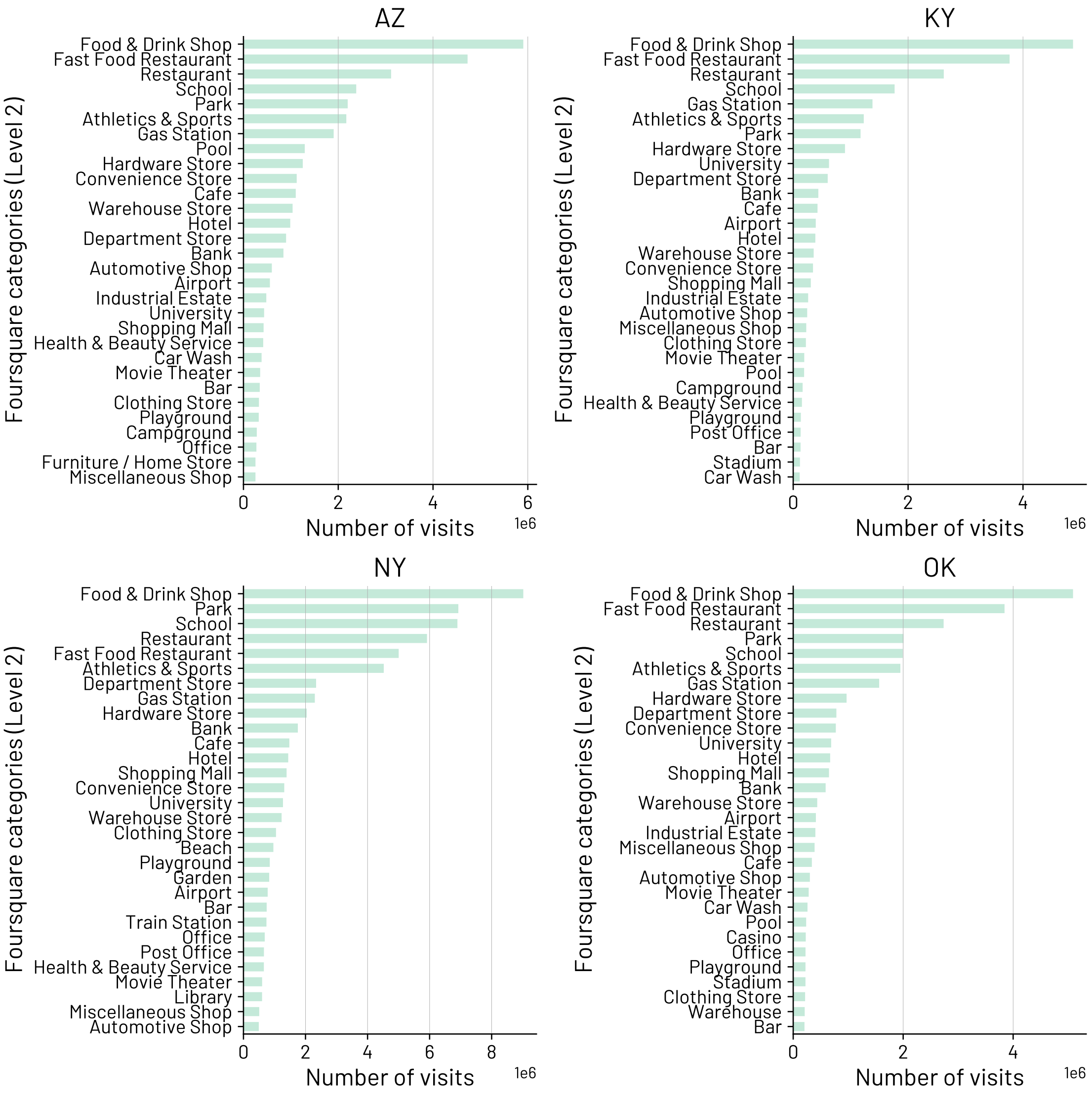

FIG. S8: Number of visits made by individuals in the four selected US states (AZ, KY, NY, OK) to POIs mapped at the Level 2 Foursquare categories. From Level 2 Foursquare categories, *health-related* categories, "Spiritual Centers", "Governmental Buildings", and "Prisons" were removed due to the sensitive nature of those locations.

D. New York state - Number and duration of visits to POIs

Here we report the number of visits and the duration of visits to POIs over time for individuals in the state of New York. Figure S9 shows these quantities for the *Arts & Entertainment*, *College & University*, *Food*, *Nightlife Spot* business venues, while Fig. S10 shows the same quantities for the *Outdoors & Recreation*, *Professional & Other Places*, *Shop & Service*, and *Travel & Transport* venue categories.

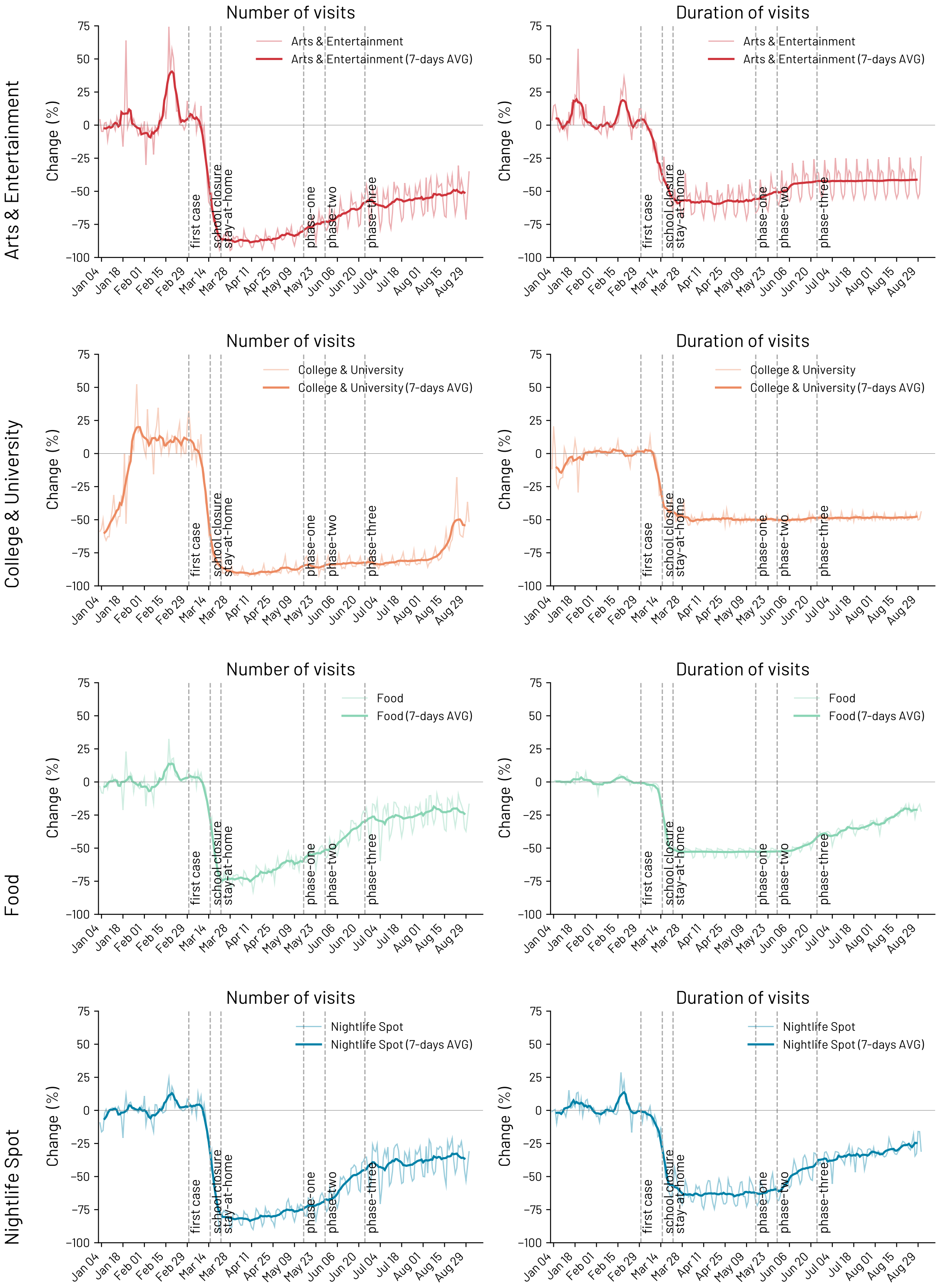

FIG. S9: Number of visits and duration of visits to POIs categories in the state of New York

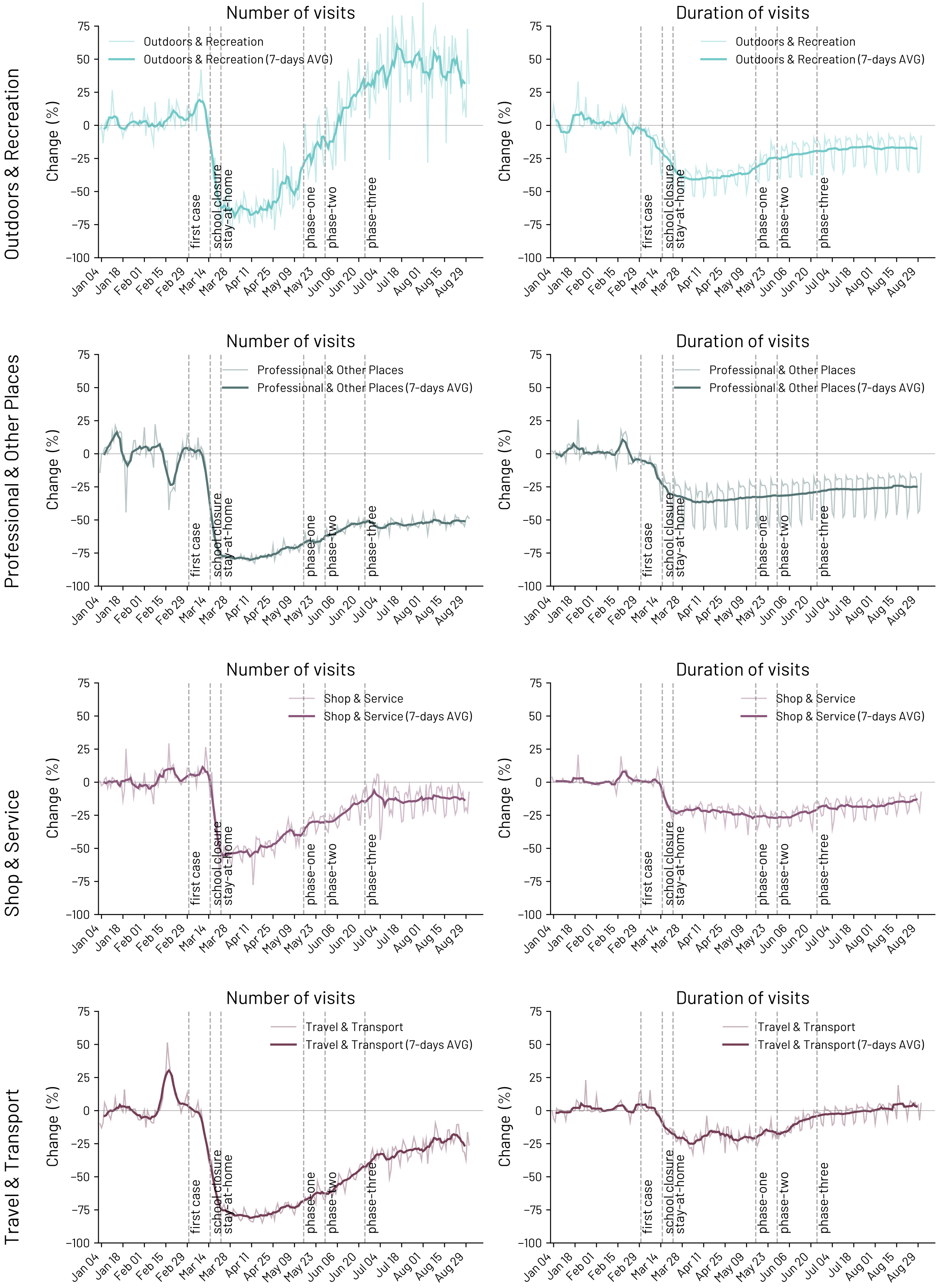

FIG. S10: Number of visits and duration of visits to POIs categories in the state of New York

E. Change in POIs' visits in Arizona, Kentucky and Oklahoma

Figure S11, Figure S12 and Figure S13 show the changes in the number and duration of visits to POIs in Arizona, Kentucky and Oklahoma respectively.

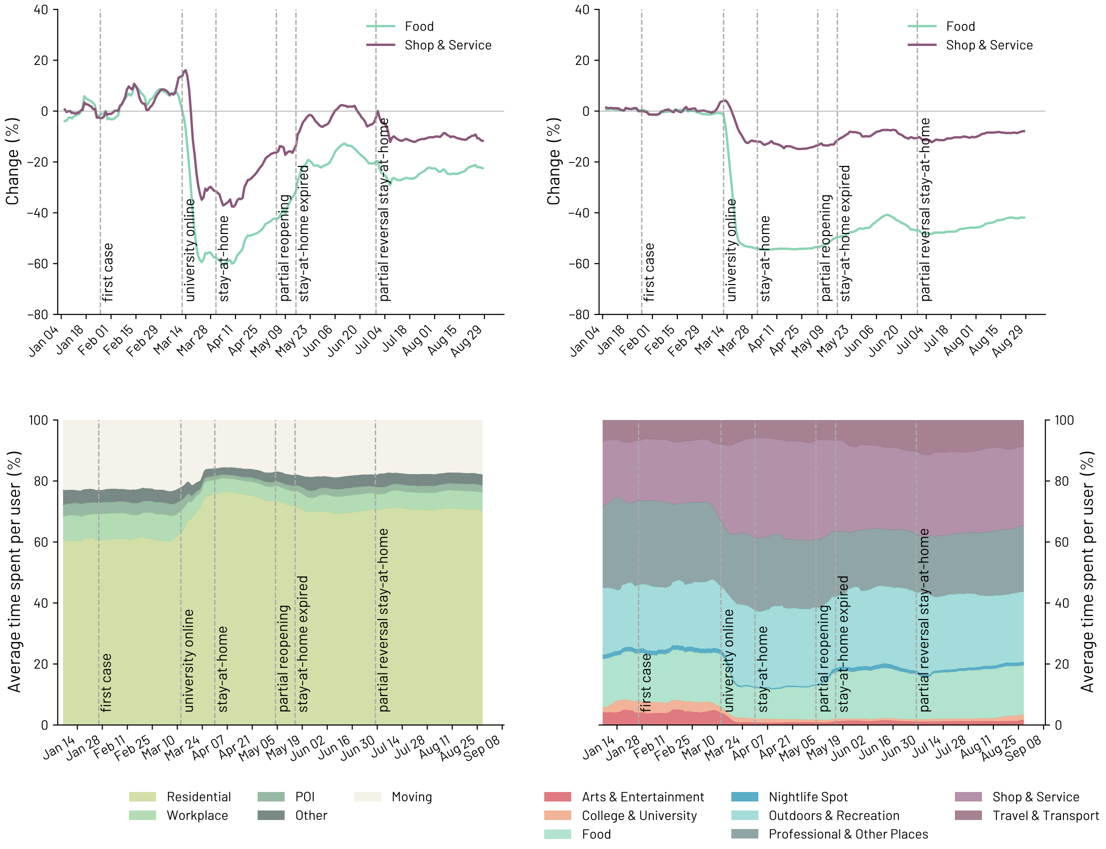

FIG. S11: Changes in number and duration of visits to POIs in the state of Arizona. A) Percent change over time in the number of visits with respect to the baseline period (January 3, 2020 - February 28, 2020) for venues in the *Food* and *Shop & Service* categories. B) Change in percentage of the duration of visits over time with respect to the baseline period of the median duration of visits to *Food* and *Shop & Service* POIs. For visualization purposes, the original curves are smoothed using a rolling average of seven days. Vertical dashed lines indicate the date of restrictions and orders imposed by the state of Arizona government. C) Percentage of time spent by people at *Residential*, *Work*, *POIs*, *Other*, and *Moving* (i.e. people in movement). D) Percentage of time spent by people in venues under the eight first-level categories of POIs.

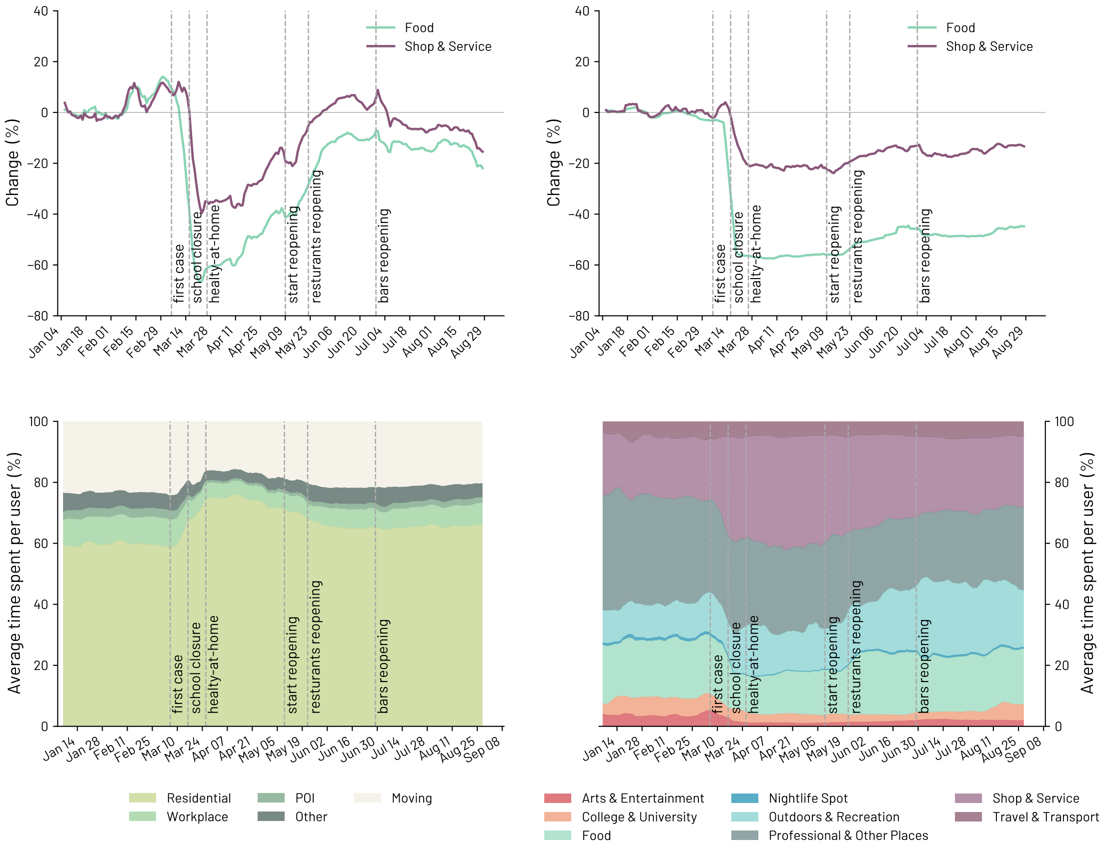

FIG. S12: Changes in number and duration of visits to POIs in the state of Kentucky. A) Percent change over time in the number of visits with respect to the baseline period (January 3, 2020 - February 28, 2020) for venues in the *Food* and *Shop & Service* categories. B) Change in percentage of the duration of visits over time with respect to the baseline period of the median duration of visits to *Food* and *Shop & Service* POIs. For visualization purposes, the original curves are smoothed using a rolling average of seven days. Vertical dashed lines indicate the date of restrictions and orders imposed by the state of Kentucky government. C) Percentage of time spent by people at *Residential*, *Work*, *POIs*, *Other*, and *Moving* (i.e. people in movement). D) Percentage of time spent by people in venues under the eight first-level categories of POIs.

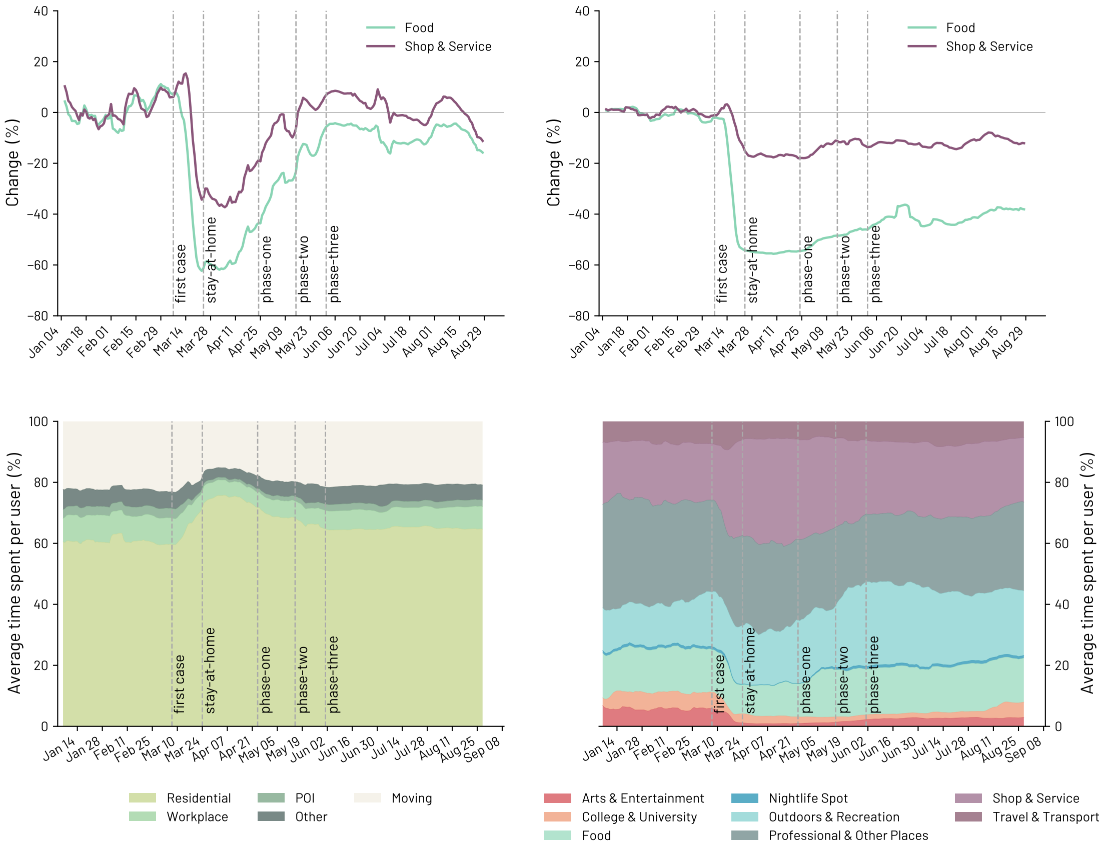

FIG. S13: Changes in number and duration of visits to POIs in the state of Oklahoma. A) Percent change over time in the number of visits with respect to the baseline period (January 3, 2020 - February 28, 2020) for venues in the *Food* and *Shop & Service* categories. B) Change in percentage of the duration of visits over time with respect to the baseline period of the median duration of visits to *Food* and *Shop & Service* POIs. For visualization purposes, the original curves are smoothed using a rolling average of seven days. Vertical dashed lines indicate the date of restrictions and orders imposed by the state of Oklahoma government. C) Percentage of time spent by people at *Residential*, *Work*, *POIs*, *Other*, and *Moving* (i.e. people in movement). D) Percentage of time spent by people in venues under the eight first-level categories of POIs.

F. Essential vs non-essential Shop & Service

We compare the time series of the number and duration of the visits to POIs between non-essential and essential *Shop & Services*. We define as essential *Shop & Services* all those POIs belonging to the *Shop & Services* category and with a second-level classification belonging to one of the following categories: *Food & Drink Shop*, *Miscellaneous Shop*, *Fruit & Vegetable Store*, *Market*, *Pharmacy*, *Miscellaneous Shop*, *Warehouse store*, *Supplement shop*, *Drugstore*, *Convenience Store*, or *Big Box Store*.

Figure S14 shows that just before the pandemic, stay-at-home orders sparked a 27% increase of visits to essential *Shop & Service* POIs, while non-essential POIs had a lower increase. During the pandemic, essential *Shop & Service* POIs faced a lower reduction than other POIs.

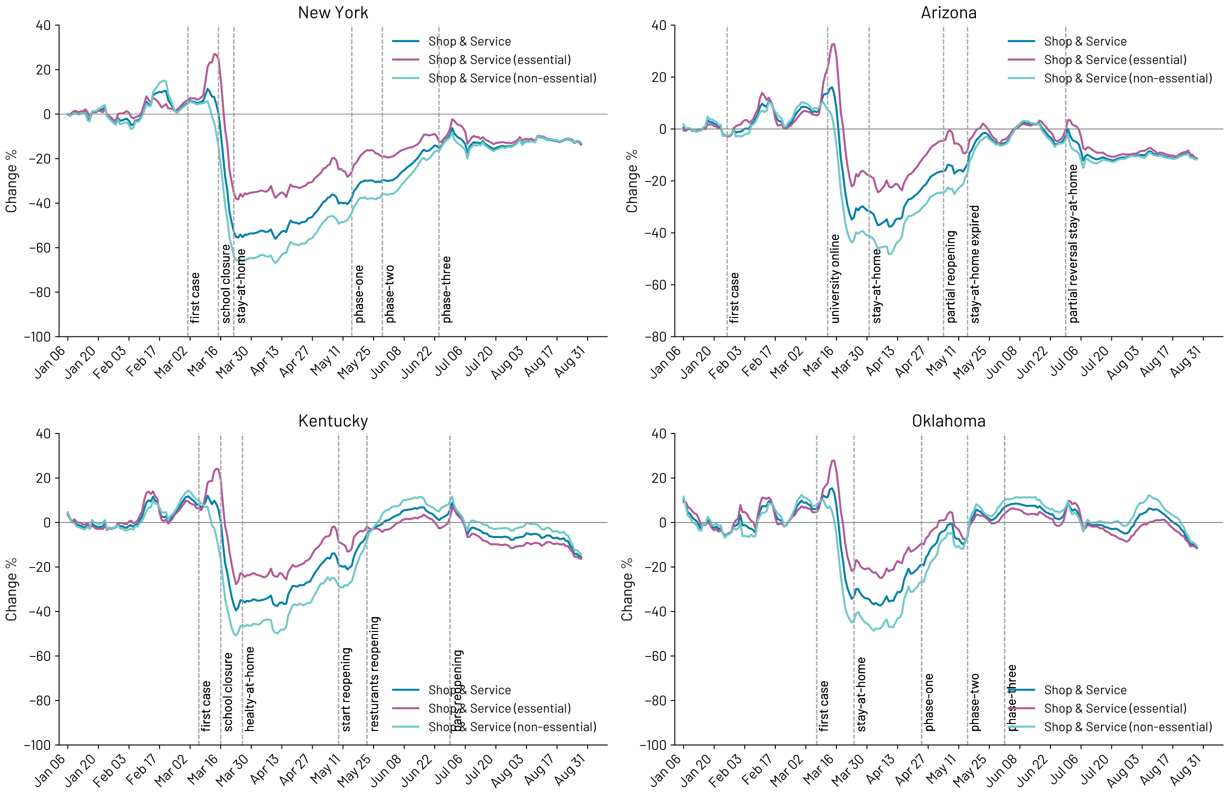

FIG. S14: Comparison of the number of visits for POIs that are essential and non-essential in the *Shop & Service* category for the selected four states, namely AZ, KY, NY, OK.

S8. COMPARISON WITH GOOGLE AND FOURSQUARE MOBILITY REPORTS

To assess the reliability of the Cuebiq data, we compared the mobility data of Cuebiq to the mobility reports provided by Google and Foursquare.

Google provides the relative change in the number of visits to a specific category by comparing the actual number of visits to a baseline computed for each day of the week, over 5 weeks from 3 January to 6 February 2020 (e.g., 5 values for Monday, 5 values for Tuesday). The only exception is for residential places, for which Google computes the change metric in terms of the fraction of time spent at home on a specific day.

We apply the same procedure to our mobility data, and we compare the results with the data provided by Google (comparing residential and workplaces) and Foursquare (comparing the different visits to POIs categories).

We computed the Pearson correlation between the relative change in the number of visits for each US state and category according to Google/Foursquare and Cuebiq.

The reliability of the Cuebiq mobility data is shown in Fig. S15, where we can see that Cuebiq mobility data show high agreement in the changes of mobility patterns with both Google and Foursquare data. Google data are used to compare the change in time spent at Residential locations and the change in the number of visits to user workplaces. Foursquare data, in contrast, are used as a comparison to check the reliability of the change in the number of visits to Foursquare venue categories.

S9. BEHAVIOUR CHANGE METRICS

As the threat of the COVID-19 pandemic unfolds in different states, there is a consistent reduction in the population mobility. This effect is reflected at various levels in different key quantities capturing several aspects of human mobility. Thus, we extract some additional mobility metrics to complement the analysis on the changes in individual mobility patterns presented in the main paper.

As shown in Fig S16, for each individual, we computed the following quantities:

- Number of unique stop locations;
- Diversity of visited locations weighted by the number of visits to an individual's locations;
- Diversity of visited locations weighted by the time spent in an individual's locations;

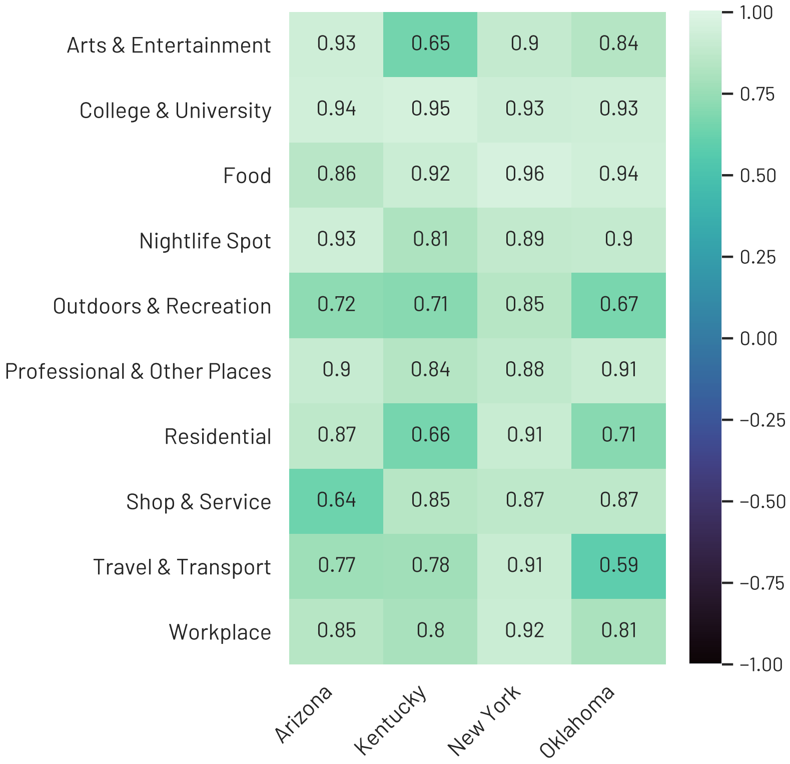

FIG. S15: Pearson correlation of the daily number of visits to POIs, number of visits to workplace, and time spent at Residential locations. Correlations are computed between Google change timeseries, for time spent at home and visits to workplaces, and the same quantities as inferred from the Cuebiq data. Comparisons for the change in number of visits to venue categories are performed by using Foursquare change visit data and the inferred visits to POI categories from Cuebiq and OpenStreetMap data.

- Radius of gyration weighted by the number of visits to an individual's locations;
- Radius of gyration weighted by the time spent in an individual's locations.

We then aggregate these metrics, averaging over all individuals. Finally, we compute the percentage change for each metric m as:

$$m_{change} = \frac{m_{w(t)} - m_b}{m_b} * 100$$

where m_b is the median of the aggregated metric m during the baseline period before the pandemic, and $m_{w(t)}$ is the median of the aggregated metric m for a window $w(t)$ for all individuals.

To compute the percentage of change for all the metrics we described, we used a time window of 2 weeks that we shift by 1 day, setting a baseline period that starts on 3 January 2020 and ends on 29 February 2020. We centre the last window at 29 February 2020, so that the last window included in the baseline period ends on 7 March 2020 (still before the stay-at-home measures).

A. Entropy of visited locations

We introduce the entropy of visited locations as a measure of the diversity of an individual's patterns of visit:

$$D_{locations}(i) = -\frac{\sum_{j=1}^{k} p_{ij} \log(p_{ij})}{\log k}.$$

Here, k is the total number of unique visited locations of an individual i, $p_{ij} = \frac{V_{ij}}{\sum_{j=1}^{k} V_{ij}}$ and V_{ij} is either (i) the number of times individual i visits location j (weighted by number of visits), or (ii) the total time individual i spends in location j (weighted by time spent).

B. Radius of gyration

The radius of gyration [6, 12] is defined as:

$$r_g = \sqrt{\frac{1}{N} \sum_{l \in L} n_l |\mathbf{r_l} - \mathbf{r_{cm}}|^2}$$

Depending on the type of weighting we apply, N is the total number of visits (or total time spent) a particular individual made to all their visited locations; n_l are the visits (time spent) to location l; L is the set of stop locations within a time window; $\mathbf{r_l}$ is a two-dimensional vector representing the location's GPS position recorded as latitude and longitude; and $\mathbf{r_{cm}}$ is the center of mass of the trajectories, defined as $\mathbf{r_{cm}} = \frac{1}{N} \sum_{l=1}^{N} \mathbf{r_l}$.

S10. MOBILITY ROUTINES

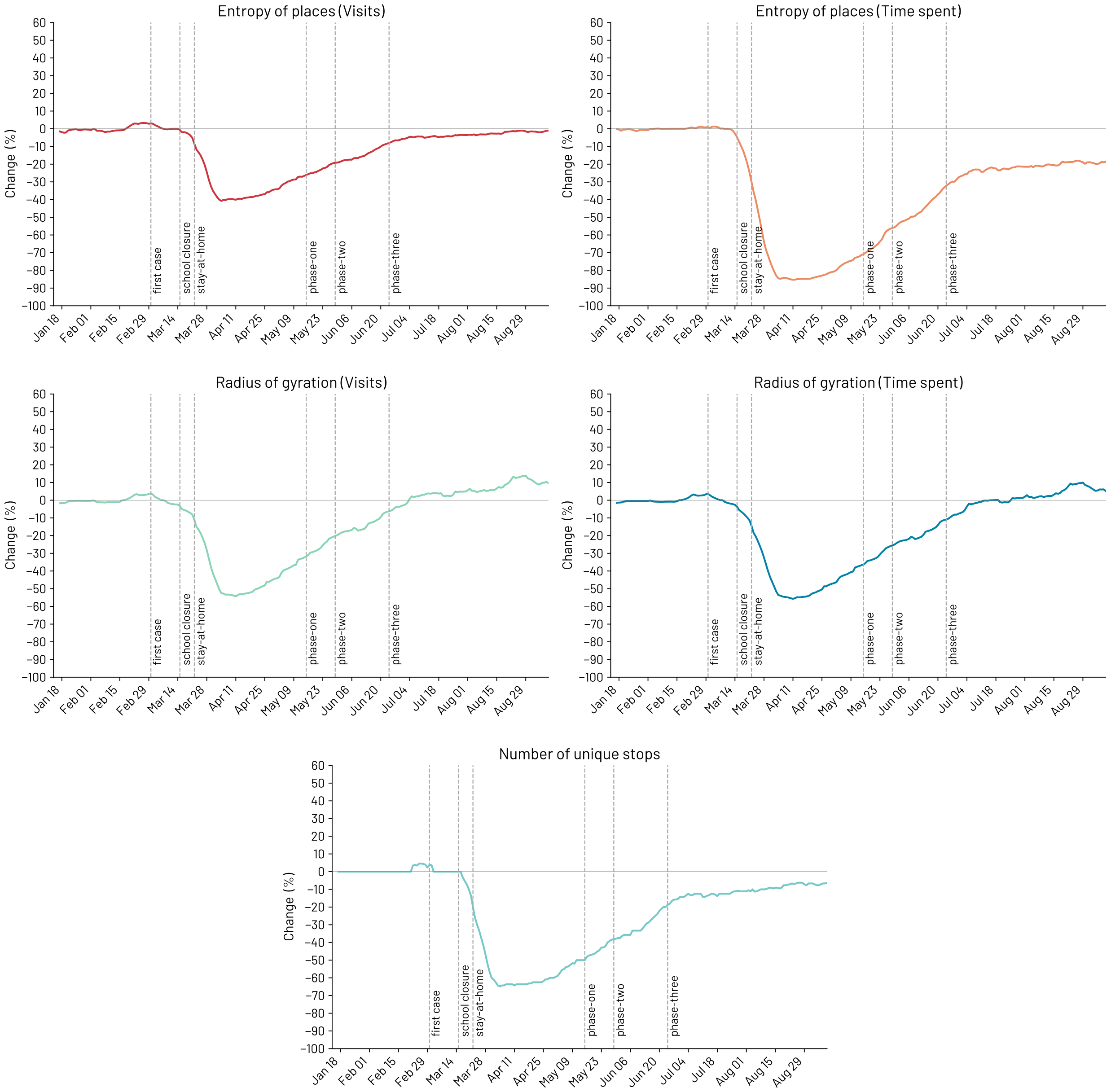

FIG. S16: Mobility behaviour change for individuals in the New York state. Median change in entropy of places (visits), entropy of places (time spent), radius of gyration (visits), radius of gyration (time spent), and number of unique stops computed on a 2-weeks window with 1-day shift

A. Comments on significant routines

The Sequitur compression algorithm is used in this work as a tool for detecting recurrent patterns in the sequence of location type visits [10]. However, since we are interested in understanding how individual routines changed as the pandemic unfolded, it is important to ensure that the recurrent patterns identified are not spurious and represent meaningful patterns in terms of individual routines'

description power. Given the nature of mobile phone data, random sequences can be erroneously identified as relevant routines. Following the approach of Di Clemente *et al.* [4], we remove routines whose number of occurrences in the original sequence, if compared to a set of randomized versions of the original sequence, does not significantly differ. This process consists of generating 1 000 copies of each individual's original sequence of visits (including their homes, workplaces, uncategorized locations, and all venue categories following the temporal order of visits) and independently shuffling each of them. We then compare the occurrences of recurrent patterns identified from the original routine with the average occurrence they show in the randomized sequences. Operationally, we compute the z-score, using each recurrent pattern standard deviation and their average number of occurrences, and keep as "significant routines" only those with a score greater than 2.

While this procedure ensures that the routines we are analyzing are representative of an individual's recurrent patterns and thus help in understanding how deeply rooted behaviours were affected by the pandemic, it is also of interest to understand the changes that occurred to the overall sequences of visits. This is performed in the next section with a thorough analysis of the direct compression of the original sequence for the two time windows under study.

B. Sequitur compression ratio

As reported in the main text, encoding individual visit patterns in a sequence of visited categories help us to better understand the extent to which human behaviour adapted to the pandemic threat under different environmental circumstances. Figure S17, Figure S18, Figure S19, and Figure S20 show the results of the compression process for the four states included in our analysis, highlighting the difference between sequences in the pre-pandemic period (from February 1, 2020, to February 28, 2020) and in the during-pandemic period (from March 25, 2020, to April 21, 2020). We find a significant reduction in sequence length from the pre-pandemic period to the during-pandemic one for all four states. This result is mirrored in the length of the compressed sequences (see panel A in Figure S17, Figure S18, Figure S19, and Figure S20). We summarize this result by computing the compression ratio, which is defined as the original sequence length, l_o, divided by the compressed sequence length, l_c, for each individual in both periods. We provide evidence of a significant increment of sequence compressibility of during-pandemic visit patterns when compared with the pre-pandemic period (under both a Welch test and Mood test at a significance level of $\alpha = 0.001$) (panels C and D). We also note that most of the non-compressible sequences (i.e. with compression ratios equal to 0) are short: during-pandemic, in 71% of the cases the length is smaller

than four. Importantly, these short sequences are mostly composed by a single stop category: during-pandemic, in 71% of the cases this category corresponds to an individual's "Residential location".

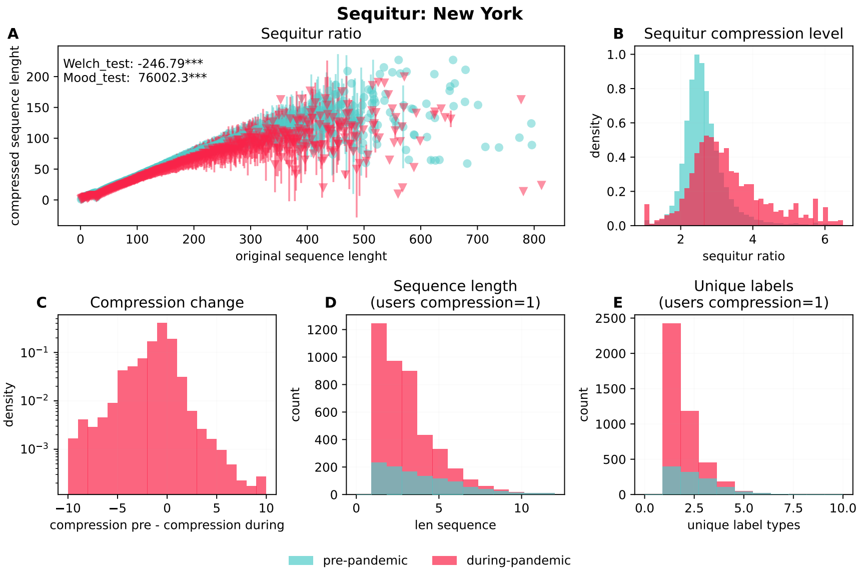

FIG. S17: Sequitur compression for individuals living in the New York state between two 28-days periods namely pre- and during-pandemic. A) scatters the median (and 95% C.I.) of compressed sequences' length as a function of their original sequence length. B) shows the distribution of the sequitur ratio while C) their element-wise difference (pre-pandemic compression ratio minus its value during-pandemic). D) and E) show the number of users with $compression\_ratio = 1$ as a function of sequence length and unique stop locations respectively.

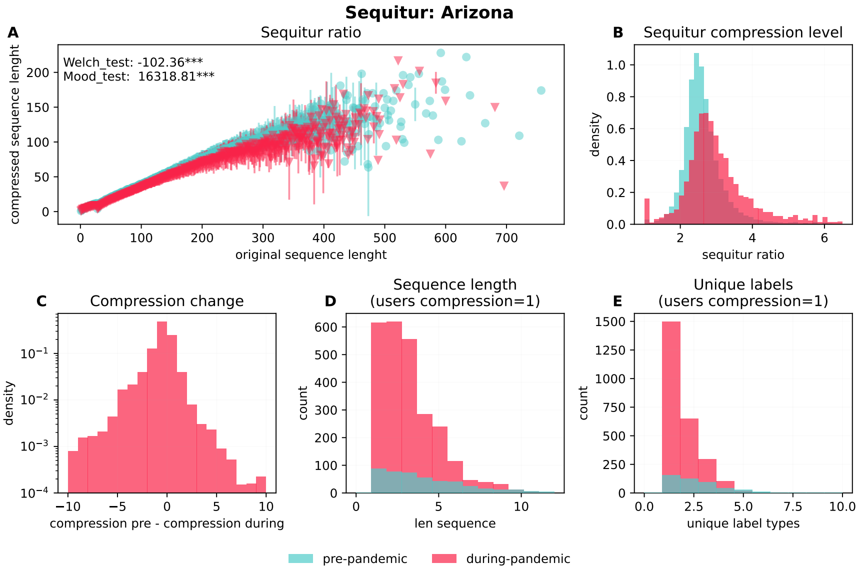

FIG. S18: Sequitur compression for individuals living in Arizona between two 28-days periods namely pre- and during-pandemic. A) scatters the median (and 95% C.I.) of compressed sequences' length as a function of their original sequence length. B) shows the distribution of the sequitur ratio while C) their element-wise difference (pre-pandemic compression ratio minus its value during-pandemic). D) and E) show the number of users with $compression\_ratio = 1$ as a function of sequence length and unique stop locations respectively.

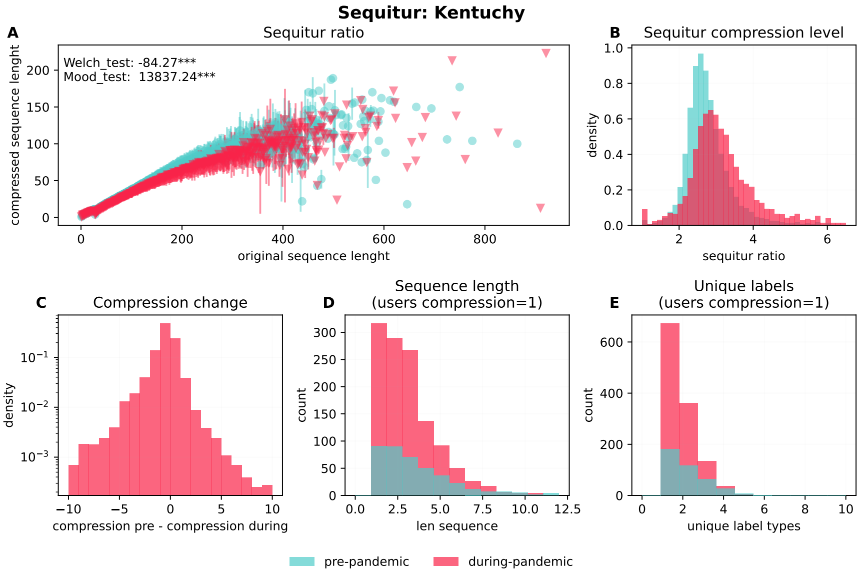

FIG. S19: Sequitur compression for individuals living in Kentucky between two 28-days periods namely pre- and during-pandemic. A) scatters the median (and 95% C.I.) of compressed sequences' length as a function of their original sequence length. B) shows the distribution of the sequitur ratio while C) their element-wise difference (pre-pandemic compression ratio minus its value during-pandemic). D) and E) show the number of users with $compression\_ratio = 1$ as a function of sequence length and unique stop locations respectively.

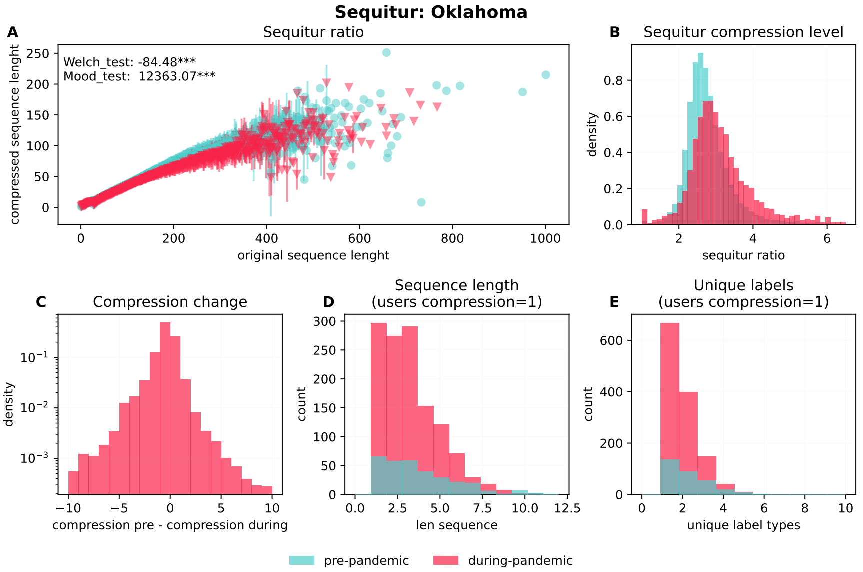

FIG. S20: Sequitur compression for individuals living in Oklahoma between two 28-days periods namely pre- and during-pandemic. A) scatters the median (and 95% C.I.) of compressed sequences' length as a function of their original sequence length. B) shows the distribution of the sequitur ratio while C) their element-wise difference (pre-pandemic compression ratio minus its value during-pandemic). D) and E) show the number of users with $compression\_ratio = 1$ as a function of sequence length and unique stop locations respectively.

C. Routine reduction

From the pre-pandemic to the during-pandemic period, we find a global decrease in all the mobility routines. In Figure S21 we report the reductions faced by each link. *College & Universities* closure exposed all the activities linked to that category to the largest reduction. A similar behaviour hit the *Arts and Entertainment* category due to the restrictions and closures of museums and other cultural places.

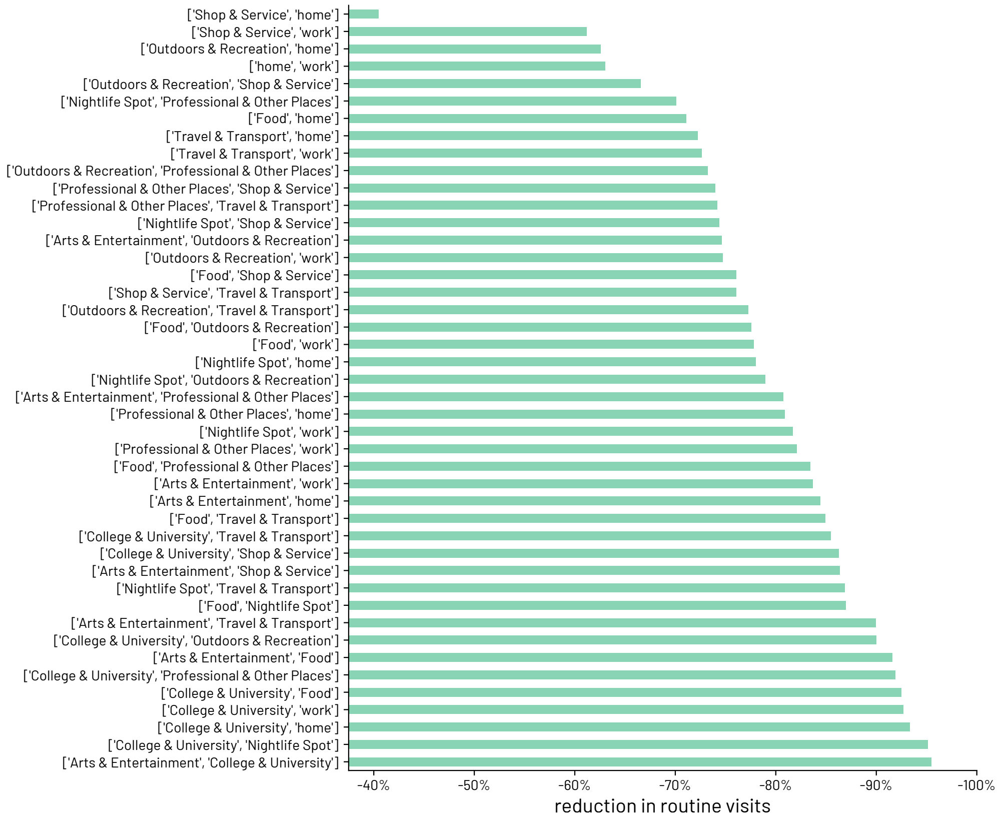

FIG. S21: Undirected routine reductions.

D. Jaccard similarity and clustering

The silhouette score is a metric that compares an object cohesion to its own cluster with its distance from other clusters. Silhouette score ranges from -1 to +1. A high value indicates a good match and in general a well matched set of cluster components. For each unit in a system, silhouette is computed as the average distance of one unit with all other units outside its own cluster $\bar{d}_{out}(i)$

minus the average distance of the same unit with the other components from its own cluster $\bar{d}_{in}(i)$:

$$s(i) = \frac{\bar{d}_{out}(i) - \bar{d}_{in}(i)}{\max \bar{d}_{out}(i), \bar{d}_{in}(i)}.$$

Figure S22 shows in panel A the average silhouette score, for the state of New York, as a function of the number of clusters as the hierarchical process progresses. As expected the clustering process is able to better group different individuals' routines during the pandemic period at all steps of the agglomerative process. Panel B shows the number of clusters as a function of the relative distance between the two elements involved in the latest clustering step. At the same distance values, we show that clustering of routines during the pandemic period have been aggregate twice as fast as during the pre-pandemic period.

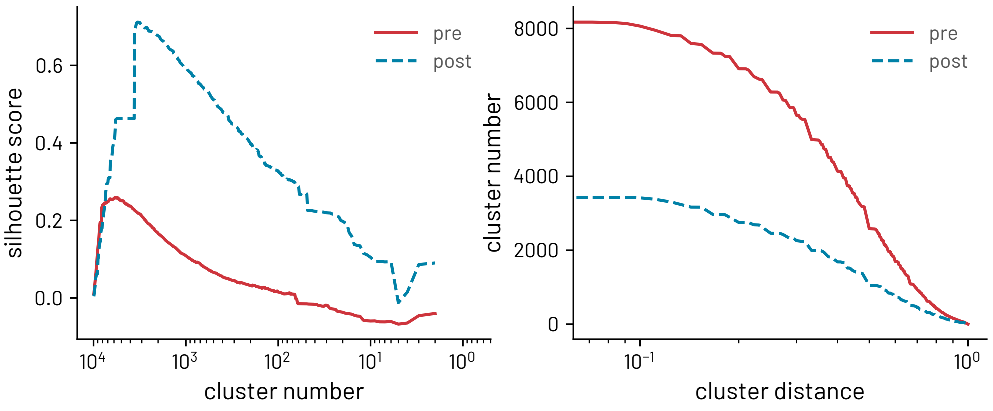

FIG. S22: Clustering pre and during the pandemic. A) shows the silhouette score as a function of the number of clusters hierarchically assembled. The red line represents the silhouette score in the pre-pandemic period, while the blue one during the pandemic. B) shows the number of clusters as a function of the largest distance between aggregated components.

E. Top clusters

To better understand the routine characteristics of people during the pandemic, we display in Figure S23 the network of subsequent visits, extracted from the top six clusters defined from the routine activity of Sequitur.

We select the clusters to visualize by selecting the top six clusters at the 95% height of the dendogram formed by the complete linkage agglomerative clustering described in the manuscript. For clarity reasons, we filter out the links with less than 5% of intensity.

Figure S23 shows that most of the users, which belong to Cluster 1 (24%) and Cluster 2 (9.6%),

have their significant locations between *Residential* and *Shops & Services*. Other clusters include more diversity in mobility, including also the work places.

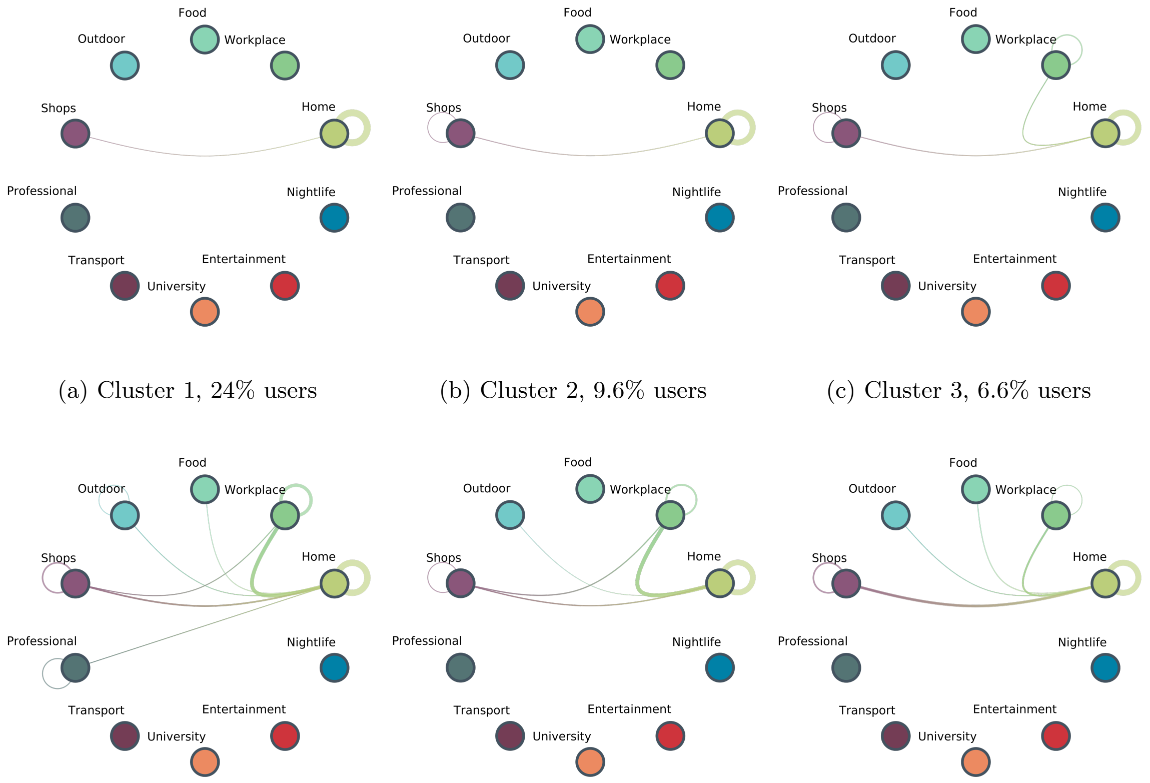

(a) Cluster 1, 24% users (b) Cluster 2, 9.6% users (c) Cluster 3, 6.6% users

(d) Cluster 4, 6.3% users (e) Cluster 5, 5.0% users (f) Cluster 6, 4.9% users

FIG. S23: The top six clusters with most individuals in the post-pandemic period.

S11. CO-LOCATION EVENTS

Two individuals are said to be "co-located" if they both have a stop location that is at most 50 meters far from the other one, and these stop locations are overlapping on date and time for at least 15 minutes. A "co-location event" is thus defined as one event in time when two or more individuals are co-located.

These *co-location events* are grouped in four different categories depending on the place where the possible social contact took place:

- *Residential*, a *co-location event* where one and only one of the two individuals have the venue marked as Residential location;

- *Workplace*, a *co-location event* that happened in a venue labeled as a workplace for both the individuals;
- *POI*, a *co-location event* where both the individuals are in the same POI (same ID of the POI);
- *Other*, a *co-location event* in which the two individuals meet in a place that it is neither a *Residential* nor a *Workplace* nor a *POI*.

A. Percent change of the number of Co-location events

Figure S24, Figure S25, Figure S26 and Figure S27 show the percent change in the number of co-location events in the states of New York, Kentucky, Oklahoma and Arizona respectively.

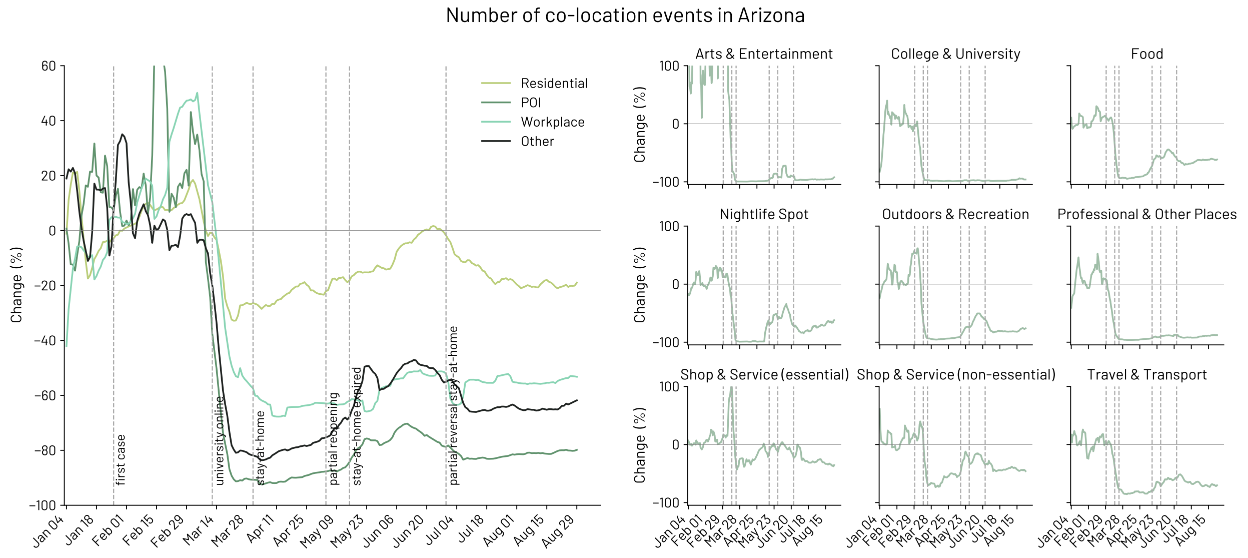

FIG. S24: Percent change in the number of co-location events from the baseline period (until 29 February 2020) in Arizona. (left) Percent change for Residential areas (one of the two individuals in proximity is at their own residential area), POI (both individuals are at the same POI), Business (both of having the same work location) and Other co-location events. (right) Percent change for all the categories of POIs.

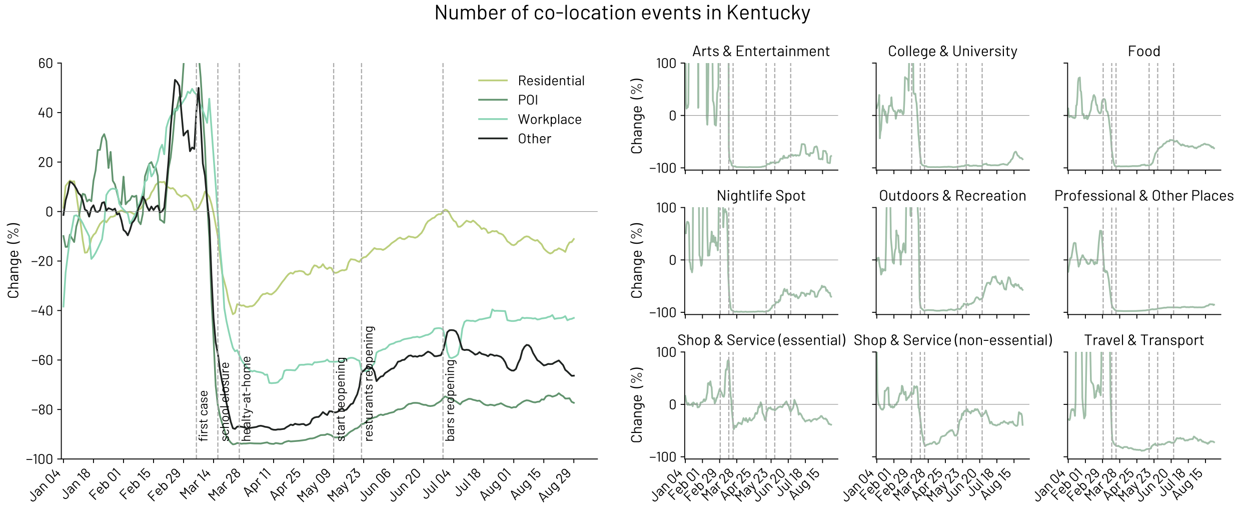

FIG. S25: Percent change in the number of co-location events from the baseline period (until 29 February 2020) in the Kentucky. (left) Percent change for Residential areas (one of the two individuals in proximity is at their own residential area), POI (both individuals are at the same POI), Business (both of having the same work location) and Other co-location events. (right) Percent change for all the categories of POIs.

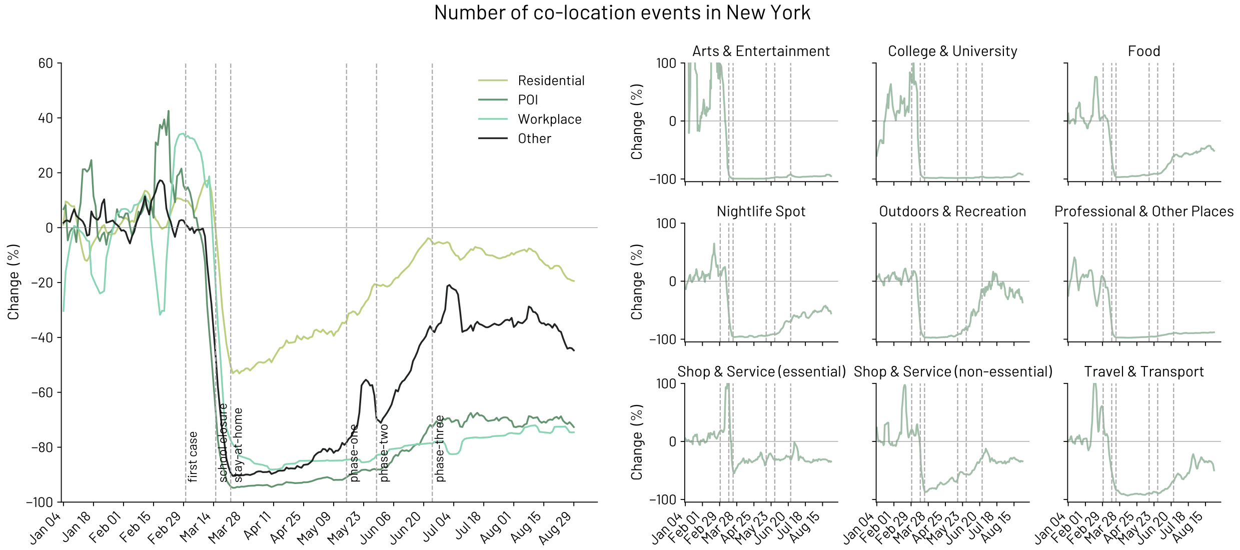

FIG. S26: Percent change in the number of co-location events from the baseline period (until 29 February 2020) in the New York. (left) Percent change for Residential areas (one of the two individuals in proximity is at their own residential area), POI (both individuals are at the same POI), Business (both of having the same work location) and Other co-location events. (right) Percent change for all the categories of POIs.

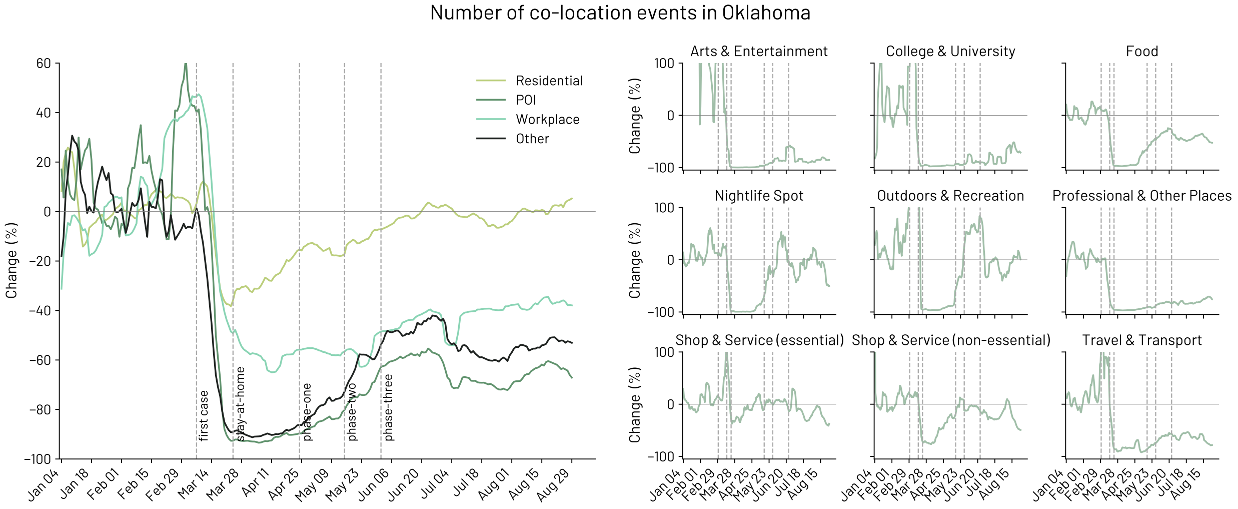

FIG. S27: Percent change in the number of co-location events from the baseline period (until 29 February 2020) in the Oklahoma. (left) Percent change for Residential areas (one of the two individuals in proximity is at their own residential area), POI (both individuals are at the same POI), Business (both of having the same work location) and Other co-location events. (right) Percent change for all the categories of POIs.

B. Percent change of the duration of Co-location events

Figure S28, Figure S29, Figure S30 and Figure S31 show the percent change in the duration of co-location events in the states of New York, Kentucky, Oklahoma and Arizona respectively.

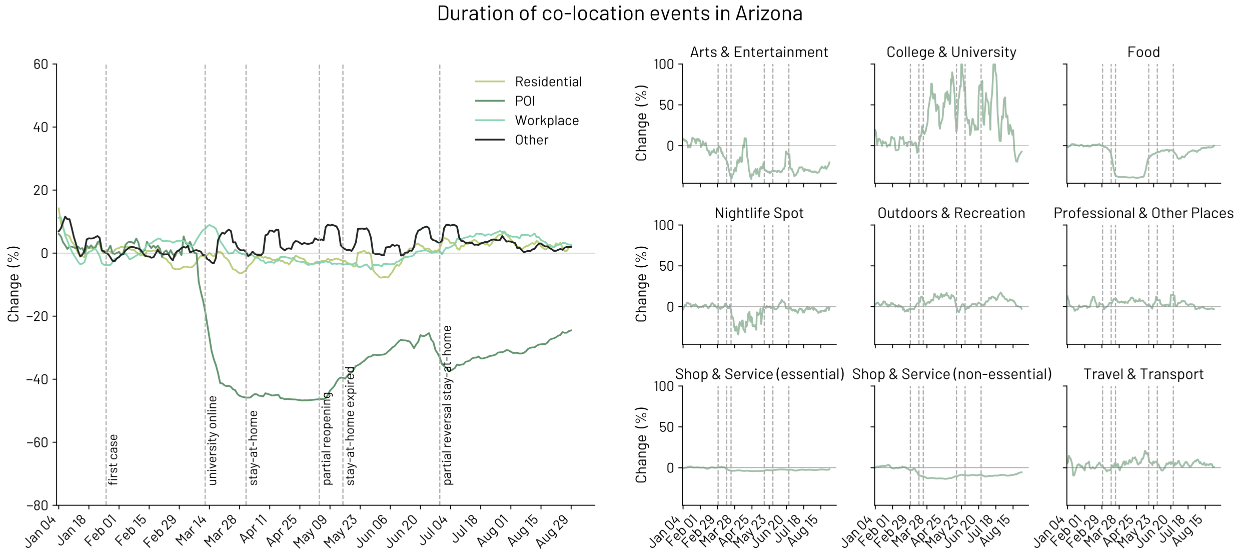

FIG. S28: Percent change of the duration of co-location events from the baseline period (until 29 February 2020) in the Arizona state. (left) Percent change for Residential areas (one of the two individuals in proximity is at their own residential area), POI (both individuals are at the same POI), Business (both of having the same work location) and Other co-location events. (right) Percent change for all the categories of POIs.

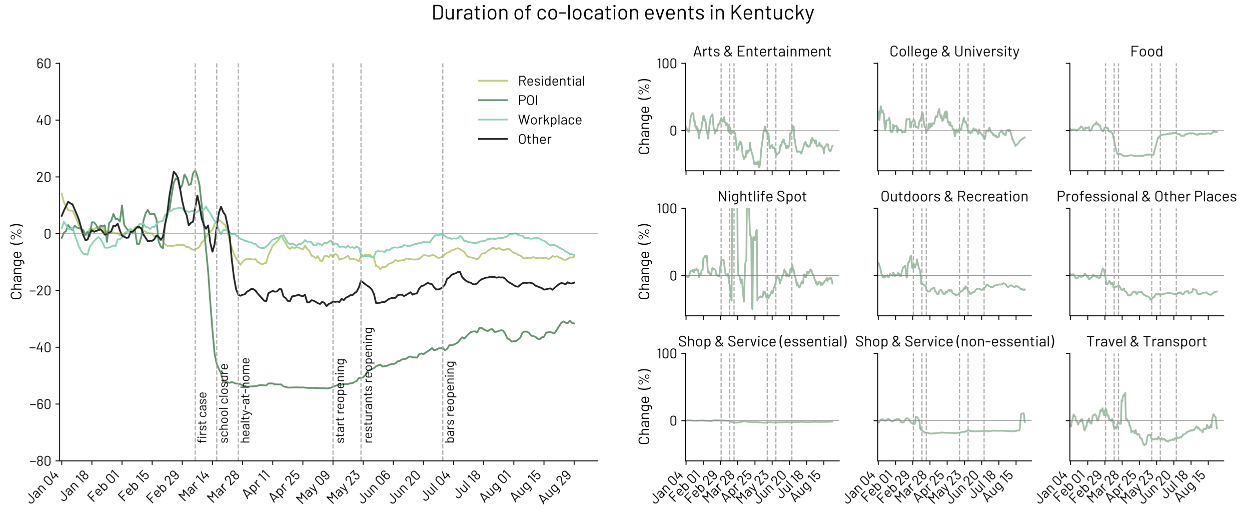

FIG. S29: Percent change of the duration of co-location events from the baseline period (until 29 February 2020) in the Kentucky state. (left) Percent change for Residential areas (one of the two individuals in proximity is at their own residential area), POI (both individuals are at the same POI), Business (both of having the same work location) and Other co-location events. (right) Percent change for all the categories of POIs.

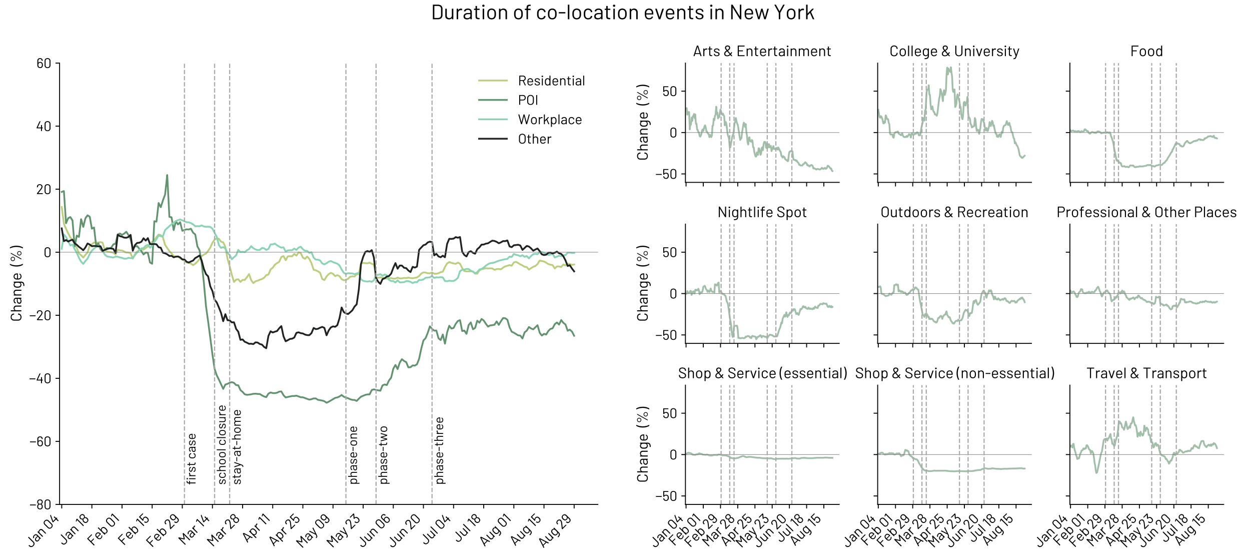

FIG. S30: Percent change of the duration of co-location events from the baseline period (until 29 February 2020) in the New York state. (left) Percent change for Residential areas (one of the two individuals in proximity is at their own residential area), POI (both individuals are at the same POI), Business (both of having the same work location) and Other co-location events. (right) Percent change for all the categories of POIs.

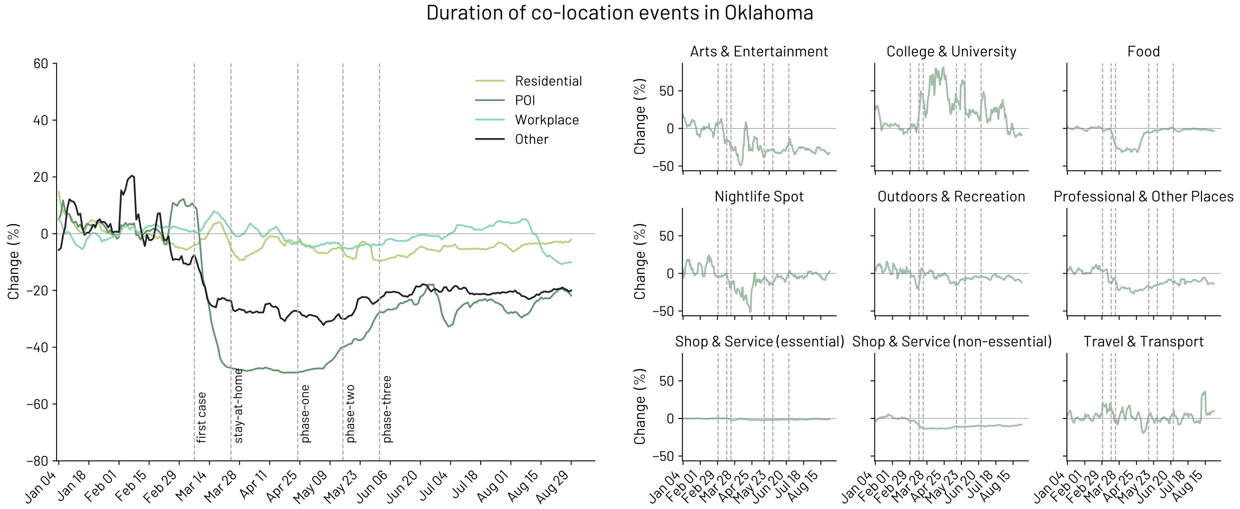

FIG. S31: Percent change of the duration of co-location events from the baseline period (until 29 February 2020) in the Oklahoma state. (left) Percent change for Residential areas (one of the two individuals in proximity is at their own residential area), POI (both individuals are at the same POI), Business (both of having the same work location) and Other co-location events. (right) Percent change for all the categories of POIs.

C. Null model

As the pandemic unfolds in the US, its impact on the number of visits to POIs grows, reducing by about 80% their number during the first phase period. This reduction of the number and duration of visits are expected to impact the number of co-location events and their duration strongly. To this extent, we propose two null models that aim at capturing the expected number and duration of co-locations given the reduction in the number of visits to POIs, the time spent at POIs, and the duration of those stops during which a co-location event occurred.

Number of co-location events null-model. To provide an estimate for the daily number of co-location events, we focus on co-locations happening at a single POI i on a specific day d. The number of co-location events occurring at that location on a specific day $e_{i,d}$ can be estimated from both the number of individuals visiting the POI $n_{i,d}$, and the median duration of their stops there $\bar{d}_{i,d}$.

We can then provide an estimate for $e_{i,d}$ using the following:

$$e_{i,d} = \binom{n_{i,d}}{2} p_{i,d} \tag{S1}$$

where $p_{i,d}$ is the probability of having a co-location event given two individuals visiting POI i on day d. $p_{i,d}$ is computed assuming a Uniform distribution for the time-interval of visit of two individuals potentially having a co-location. This means that, if two individuals visited the same POI i on day d, they will have a non-null probability of visiting i with partially (or completely) overlapping time-intervals of visit. The probability that the two intervals have an overlap of at least ϵ time is then computed as a fraction of the length of their stop duration and the duration of a day with open boundary conditions:

$$p_{i,d} = \begin{cases} 0 & \bar{d}_{i,d} < \epsilon \\ \frac{2(\bar{d}_{i,d} - \epsilon/2)}{\tilde{d}} & \epsilon \leq \bar{d}_{i,d} < \tilde{d}/2 + \epsilon/2 \\ 1 & \bar{d}_{i,d} \geq \tilde{d}/2 + \epsilon/2 \end{cases} \tag{S2}$$

where ϵ is a minimum number of minutes the two time-interval are required to overlap in order for a co-location to be counted as a co-location event, and $\tilde{d}$ is the duration of a day in minutes. The null model assumes that, if $\bar{d}_{i,d}$ is smaller than ϵ, then no co-location will be counted as a co-location event. In contrast, if $\bar{d}_{i,d}$ is greater than ϵ, the probability of a co-location events grows linearly until the minimum overlap region is equal to ϵ, namely when $\bar{d}_{i,j} = \tilde{d}/2 + \epsilon/2$. For all values greater than that value, the probability of a co-location event if the people visit the same POI is 1.

The open boundary condition ensures that the co-location overlap of one individual stop with the second is independent from the time at which the second individual visits the POI. We note that, as the median duration of POI visit increases, the co-location event probability grows linearly twice as fast.

Co-location event duration null-model. The expected duration of a co-location event is estimated following a similar reasoning. Conditioning on the fact that two individuals are co-located at POI i on day d, we use the average length of their stops there, namely $\hat{d}_{i,d}$, to estimate their expected time-overlap under a uniformly distributed probability for the displacement of their visit over the entire day. Thus, our null-model assumes that individuals visit POIs without the specific intent of meeting with someone. Under this assumption, the expected temporal overlap, $o_{i,d}$, which corresponds the expected duration of a co-location events, can be easily derived as the average overlap between two time-intervals moving over the same domain:

$$o_{i,d} = \frac{1}{2(\hat{d}_{i,d} - \epsilon)} \int_{-\hat{d}_{i,d}+\epsilon}^{\hat{d}_{i,d}-\epsilon} (\hat{d}_{i,d} - |x|) \cdot dx \tag{S3}$$

$$= \frac{\hat{d}_{i,d} + \epsilon}{2} \tag{S4}$$

where x is the distance between the center of the two intervals of equal length $\hat{d}_{i,d}$.

Figure S32, Figure S33, and Figure S34 show the percent change in: A) the number of co-location events divided per co-location event type; B) the percent change in co-location events happening in POIs (full line) against the expected co-location events (dashed line); C) the percent change in the duration (i.e. the time overlap) of co-location events happening in POIs (full line) against the expected duration of co-location events (dashed line). Figures Figure S32, Figure S33, and Figure S34 show the results for the state of Kentucky, Oklahoma and Arizona respectively.

S12. VISITS TO POIS: MODEL CONSTRUCTION AND MODEL SELECTION

This section reports all the models tested and their construction in terms of prior distributions and sampling algorithms.

Prior selection: Here, we introduce the prior distributions adopted to fit the full model. The baseline and weather models use the same prior distribution without the additional parameters introduced in the full model only. Therefore, for the sake of simplicity, we report here the complete list of priors without differentiating for the three main models presented and discussed in the main

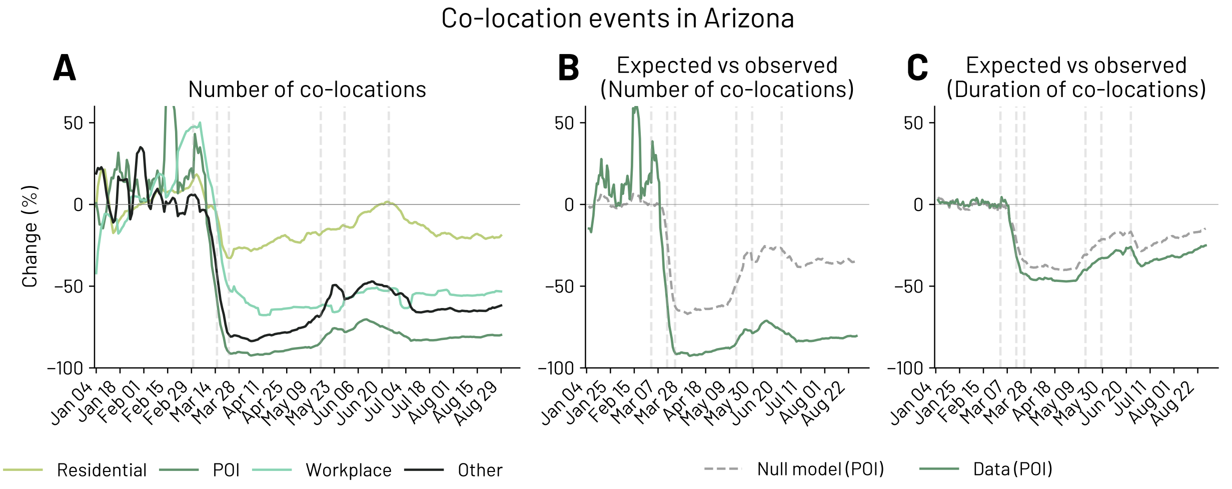

FIG. S32: A) Percentage change of co-location events from the baseline period (until 29 February 2020) in the Arizona state. We measure the change for Residential areas (one of the two individuals in proximity is at their own residential area), POI (both individuals are at the same POI), Business (both of having the same work location) and Other co-location events. B-C) We compare the difference between the expected and observed number and duration of co-location events. The figure shows that during the pandemic, individuals tend to have less co-locations than expected. However, it seems that co-locations are longer than expected.

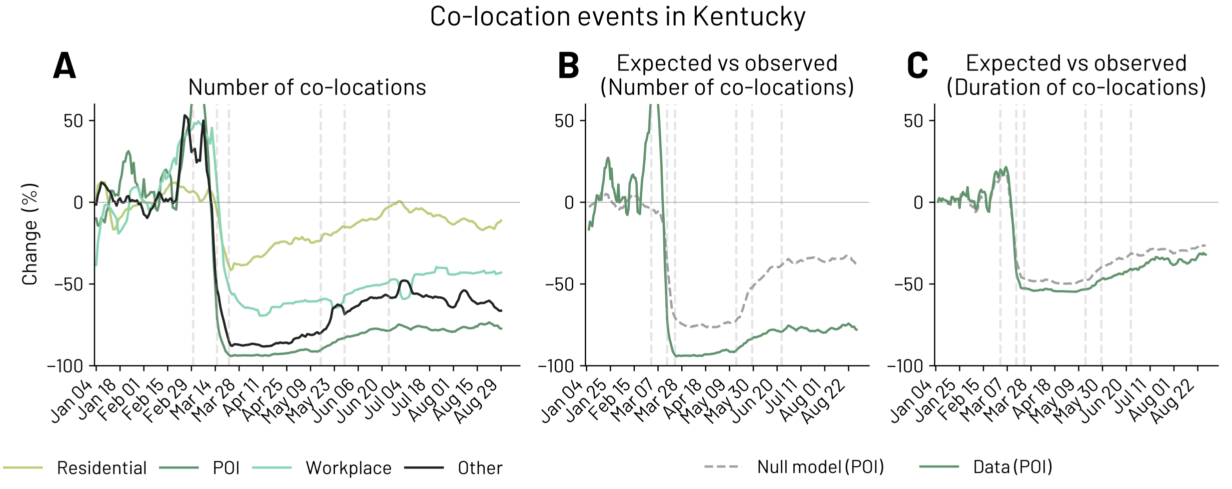

FIG. S33: A) Percentage change of co-location events from the baseline period (until 29 February 2020) in the Kentucky state. We measure the change for Residential areas (one of the two individuals in proximity is at their own residential area), POI (both individuals are at the same POI), Business (both of having the same work location) and Other co-location events. B-C) We compare the difference between the expected and observed number and duration of co-location events. The figure shows that during the pandemic, individuals tend to have less co-locations than expected. However, it seems that co-locations are longer than expected.

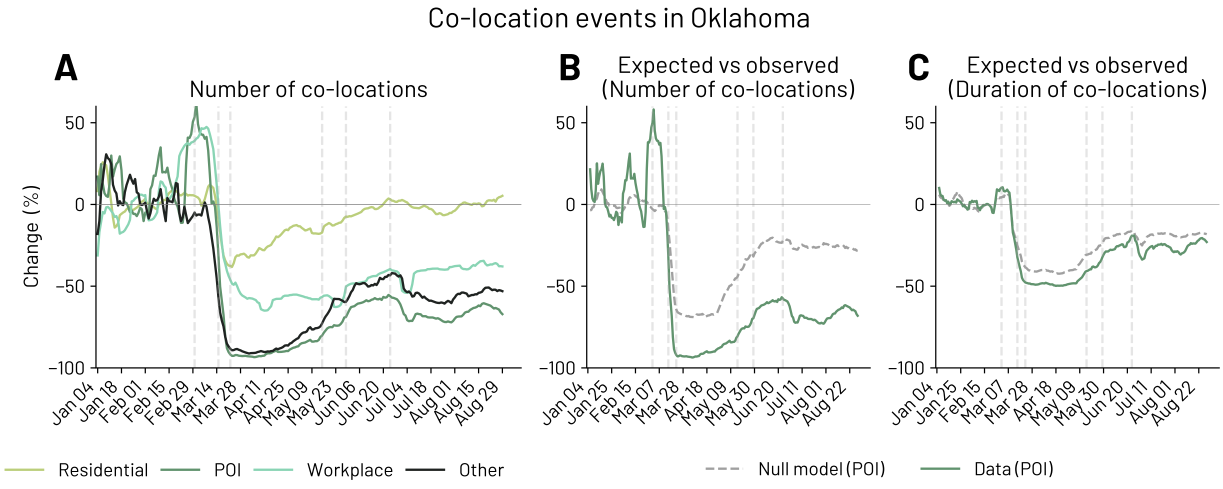

FIG. S34: A) Percentage change of co-location events from the baseline period (until 29 February 2020) in Oklahoma state. We measure the change for Residential areas (one of the two individuals in proximity is at their residential area), POI (both individuals are at the same POI), Business (both of having the same work location) and Other co-location events. B-C) We compare the difference between the expected and observed number and duration of co-location events. The figure shows that during the pandemic, individuals tend to have fewer co-locations than expected. However, it seems that co-locations are longer than expected.

paper.

$$\begin{aligned}
\alpha_s &\sim Normal(\mu = 0, \sigma = 1)\\
\beta_{policy} &\sim Normal(\mu = -1, \sigma = 0.1)\\
\beta_{deaths} &\sim Normal(\mu = -1, \sigma = 0.1)\\
\rho_i &\sim Normal(\mu = 0, \sigma = 0.1)\\
\beta_{temp} &\sim Normal(\mu = 0, \sigma = 0.1)\\
\beta_{prec} &\sim Normal(\mu = 0, \sigma = 0.1)\\
\beta_{adapt} &\sim HalfNormal(\sigma = 0.1)\\
\gamma_s &\sim 0.01 + HalfNormal(\sigma = 0.1)\\
\phi_s &\sim 90 + HalfNormal(\sigma = 10)\\
\epsilon &\sim HalfNormal(\sigma = 0.05)\\
Y &\sim Student(\mu = y_{model}, \sigma = \epsilon, \nu = 3).
\end{aligned}$$

Cumulated stringency model: Our research question aims at understanding if a model accounting for a behavioural adaptation to risk after a sustained period of exposure can better describe

model	metric	loo	se	r2	r2std	weight
**full**	mean visits	846.770	29.598	0.783	0.024	0.999
**cumulated strin.**	mean visits	811.043	26.941	0.758	0.017	0.001
**weather**	mean visits	747.450	28.780	0.728	0.019	0.000
**baseline**	mean visits	610.603	31.943	0.674	0.022	0.000

TABLE S2: **Summary results for the models describing the average number of visits per individual per day.** Results show that the full model outperforms all the other in terms of the Pareto-smoothed importance sampling Leave-One-Out cross-validation information criterion (PSIS-LOO) [14]. The *cumulated Stringency* model following a similar idea as the full model shows better performances if compared with the weather and the baseline models.

model	metric	loo	se	r2	r2std	weight
**full**	time not in residential areas	328.691	42.865	0.858	0.013	1.000
**cumulated strin.**	time not in residential areas	234.250	37.602	0.820	0.018	0.000
**weather**	time not in residential areas	192.226	40.513	0.807	0.018	0.000
**baseline**	time not in residential areas	138.522	46.064	0.797	0.020	0.000

TABLE S3: **Summary results for the models describing the average time spent outside the residential area per individual per day.** Results show that the full model outperforms all the other in terms of the Pareto-smoothed importance sampling Leave-One-Out cross-validation information criterion (PSIS-LOO) [14]. The *cumulated Stringency* model, following a similar idea as the full model, shows better performances if compared with the weather and the baseline models.

the individual's tendency to visit POIs or not. The formulation of the full model takes this effect into account by adding a dependency on the time elapsed after the first pandemic restrictions. To confirm and test the robustness of the full model results, we also test for a different model, namely the "cumulated stringency model", which instead of adding a dependency on time, uses the value of the cumulated restrictions intensity as a proxy of the pandemic burden experienced so far. Table S12 shows that also this model better captures the reduction and subsequent recovery of the number of visits to POIs.

Modeling "time not at home" : As an additional robustness check, we also tested the performances of these four models in describing the timeseries of the time not spent in residential areas. Also in this case, Tab. S12 summarizes the better performance of both models accounting for a behavioural adaptation component.

[1] Foursquare 2020. Venue categories — build with foursquare. Accessed on 2021-08-17.

[2] Bureau of Labor Statistics, American Time Use Survey. Percent of population who worked on weekdays and weekend days. `https://www.bls.gov/tus/charts/chart11.pdf`, 2015.

[3] Cuebiq. Sensitive points of interest policy - cuebiq. Accessed on 2021-07-22.

[4] Riccardo Di Clemente, Miguel Luengo-Oroz, Matias Travizano, Sharon Xu, Bapu Vaitla, and Marta C González. Sequences of purchases in credit card data reveal lifestyles in urban populations. *Nature communications*, 9(1):1–8, 2018.

[5] Martin Ester, Hans-Peter Kriegel, Jörg Sander, and Xiaowei Xu. A density-based algorithm for discovering clusters a density-based algorithm for discovering clusters in large spatial databases with noise. In *Proceedings of the Second International Conference on Knowledge Discovery and Data Mining*, KDD'96, pages 226–231. AAAI Press, 1996.

[6] Marta C Gonzalez, Cesar A Hidalgo, and Albert-Laszlo Barabasi. Understanding individual human mobility patterns. *nature*, 453(7196):779–782, 2008.

[7] Thomas Hale, Noam Angrist, Rafael Goldszmidt, Beatriz Kira, Anna Petherick, Toby Phillips, Samuel Webster, Emily Cameron-Blake, Laura Hallas, Saptarshi Majumdar, et al. A global panel database of pandemic policies (oxford covid-19 government response tracker). *Nature Human Behaviour*, 5(4):529–538, 2021.

[8] Ramaswamy Hariharan and Kentaro Toyama. Project lachesis: Parsing and modeling location histories. In Max J. Egenhofer, Christian Freksa, and Harvey J. Miller, editors, *Geographic Information Science*, pages 106–124, Berlin, Heidelberg, 2004. Springer Berlin Heidelberg.

[9] Shan Jiang, Yingxiang Yang, Siddharth Gupta, Daniele Veneziano, Shounak Athavale, and Marta C González. The timegeo modeling framework for urban mobility without travel surveys. *Proceedings of the National Academy of Sciences*, 113(37):E5370–E5378, 2016.

[10] Craig G Nevill-Manning and Ian H Witten. Identifying hierarchical structure in sequences: A linear-time algorithm. *Journal of Artificial Intelligence Research*, 7:67–82, 1997.

[11] OxCGRT. covid-policy-tracker/codebook.md · oxcgrt/covid-policy-tracker · github. Accessed on 2021-06-25.

[12] Luca Pappalardo, Filippo Simini, Salvatore Rinzivillo, Dino Pedreschi, Fosca Giannotti, and Albert-László Barabási. Returners and explorers dichotomy in human mobility. *Nature communications*, 6(1):1–8, 2015.

[13] Henrikki Tenkanen + pyrosm contributors. Pyrosm. Accessed on 2021-01-29.

[14] Aki Vehtari, Andrew Gelman, and Jonah Gabry. Practical bayesian model evaluation using leave-one-out cross-validation and waic. *Statistics and computing*, 27(5):1413–1432, 2017.

[15] Wikipedia. Business hours - wikipedia. Accessed on 2021-06-21.

[16] Wikipedia. Map features - openstreetmap wiki. Accessed on 2021-09-13.

[17] Wikipedia. U.s. state and local government responses to the covid-19 pandemic - wikipedia. Accessed on 2021-07-01.
